# 3D particle averaging and detection of macromolecular symmetry in localization microscopy

**DOI:** 10.1038/s41467-021-22006-5

**Published:** 2021-05-14

**Authors:** Hamidreza Heydarian, Maarten Joosten, Adrian Przybylski, Florian Schueder, Ralf Jungmann, Ben van Werkhoven, Jan Keller-Findeisen, Jonas Ries, Sjoerd Stallinga, Mark Bates, Bernd Rieger

**Affiliations:** 1 Department of Imaging Physics, Delft University of Technology, Delft, The Netherlands; 2 Department of NanoBiophotonics, Max Planck Institute for Biophysical Chemistry, Göttingen, Germany; 3 Department of Physics and Center for Nanoscience, Ludwig Maximilian University, Munich, Germany; 4 Max Planck Institute of Biochemistry, Martinsried, Germany; 5 Netherlands eScience Center, Amsterdam, The Netherlands; 6 Cell Biology and Biophysics Unit, European Molecular Biology Laboratory (EMBL), Heidelberg, Germany

**Keywords:** Super-resolution microscopy, Structure determination

## Abstract

Single molecule localization microscopy offers in principle resolution down to the molecular level, but in practice this is limited primarily by incomplete fluorescent labeling of the structure. This missing information can be completed by merging information from many structurally identical particles. In this work, we present an approach for 3D single particle analysis in localization microscopy which hugely increases signal-to-noise ratio and resolution and enables determining the symmetry groups of macromolecular complexes. Our method does not require a structural template, and handles anisotropic localization uncertainties. We demonstrate 3D reconstructions of DNA-origami tetrahedrons, Nup96 and Nup107 subcomplexes of the nuclear pore complex acquired using multiple single molecule localization microscopy techniques, with their structural symmetry deducted from the data.

## Abstract (web summary)

Adaptation of current algorithms to 3D SMLM data is currently problematic. Here the authors report a method that increases the signal-to-noise ratio and resolution of 3D single particle analysis in localization microscopy and enables determination of the symmetry groups of macromolecular complexes.

## Introduction

Single molecule localization microscopy (SMLM) is one of the most widely applied types of optical super-resolution microscopy. The image resolution is ultimately limited by the density of the fluorescent labels on the structure of interest and the finite precision of each localization^1,2^. Recently, methods for obtaining higher precision localizations have been reported, which work by either increasing the number of collected photons per molecule via e.g. cryogenic imaging^3,4^, or by introducing patterned illumination^5,6^. The first limitation remains, however, and one approach to boosting the apparent degree of labeling (DOL) and filling in missing labels can be applied when the sample consists of many identical copies of the structure of interest (e.g. a macromolecule). In this case, combining many structures into a single “super-particle” increases the effective labeling density and improves the signal-to-noise ratio (SNR) and resolution significantly. Besides these improvements, structural features of the data such as symmetry give insight into the morphology and functional properties of subcellular structures. In SMLM, this has been limited so far to the detection of rather simple morphologies^7^, but no algorithms have been introduced that can find arbitrary symmetry group(s) needed to characterize 3D structures.

Existing approaches to particle averaging in SMLM can be classified as either template-based or as adaptations of single particle analysis (SPA) algorithms for cryo-electron microscopy (EM) images. Template-based methods^8,9^ are computationally efficient, but are susceptible to template bias artefacts. Methods derived from SPA for cryo-EM^10,11^ have been employed to generate 3D reconstructions from 10^4^ to 10^6^ 2D projections of random viewing angles of a structure. However, there are two major problems with the adaptation of these algorithms to 3D SMLM data. Firstly, the image formation in cryo-EM^9^ differs from SMLM where in the first the electron-specimen interaction potential is imaged (a continuous function) and in the latter (repeated) localizations of a fluorescently (under) labeled structure are imaged. Secondly, the inherent 2D nature of the input data. While the first problem can be ignored in favorable experimental conditions such as high labeling density, high localization precision, and abundant number of localizations, the latter problem remains. The three-dimensional data of 3D SMLM (*x*, *y*, *z* coordinates) is not compatible with 2D processing even if you render the localizations into a voxelated representation. Of course projecting the data to 2D would unnecessarily throw away information and increase the problem of pose estimation. Sub-tomogram averaging^12^ utilizes the 3D tomographic reconstruction primarily to identify the particle locations but the actual averaging and final reconstruction is again done on the 2D projections as in SPA to avoid missing wedge reconstruction artifacts present in the tomogram. Recently, Shi et. al.^13^ also described a structure-specific method for 3D fusion, although they implicitly assume cylindrical particles and projected the volume onto top views only.

Here, we introduce a 3D particle fusion approach for SMLM which does not require, but can incorporate, a priori knowledge of the target structure such as the symmetry group. It works directly on 3D volume of localization data, rather than 2D projections, and accounts for anisotropic localization uncertainties. In addition, we propose a method for detecting the full rotational symmetry group of the structure from the data itself, which can subsequently be used in order to improve the fusion outcome. We report 3D reconstructions of the Nuclear Pore Complex (NPC) obtained from three different SMLM techniques. The results demonstrate a two orders of magnitude SNR amplification, and Fourier shell correlation (FSC) resolution values as low as 14–16 nm, which enables the structural identification of distinct proteins within a large macromolecular complex such as the NPC. We further retrieve the 8-fold rotational symmetry of the NPC assembly and the full tetragonal symmetry of a 3D tetrahedron DNA-origami nanostructure, without any prior knowledge imposed on the data.

## Results

### 3D particle fusion pipeline

**Fig. 1:**
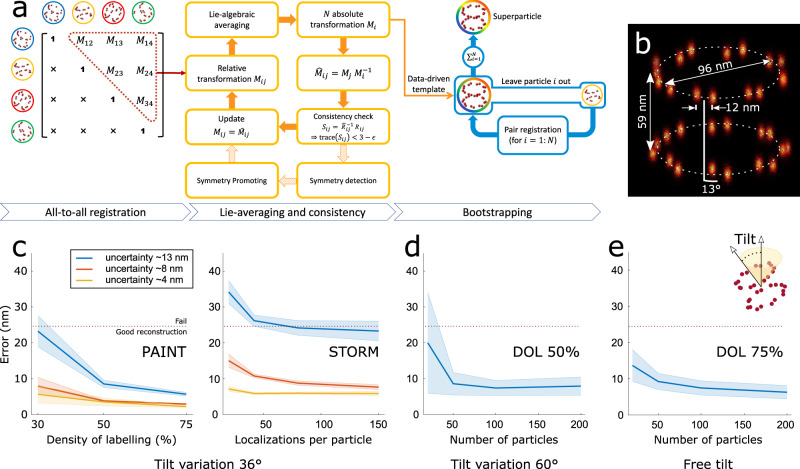
The 3D SMLM particle fusion pipeline and results of the simulation study. **a** Pair registration of all segmented particles results in relative transformations *M*_*ij*_ (translations *t*_*ij*_ and rotations *R*_*ij*_). The redundant information in the all-to-all registration matrix is utilized for improving the registration errors by means of Lie-algebraic averaging, which results in *M*_*i*_ absolute transformations. The relative transformations are recomputed as *M*_*j*_*M*_*i*_^−1^. From them, a consistency check (based on only rotations *R*_*ij*_) is applied via a threshold ε on the rotation error to remove outlier registrations *M*_*ij*_ from the all-to-all matrix. After two iterations, this results in a data-driven template. Additionally, the rotation error residuals that are encoded in the histogram of *S*_*ij*_ can be used to infer symmetry group(s) of the particle structure and to subsequently impose symmetry on the data. Finally, five rounds of bootstrapping are applied to improve the final reconstruction by registering every particle to the derived template. **b** Ground-truth fusion of 100 simulated NPCs indicating the height, radius, the angular shift between the cytoplasmic and nuclear rings in the same NPC. **c** Registration error for simulated PAINT and STORM data for different degree of labeling (DOL), mean localization uncertainties (*σ* = 4, 8, and 13 nm) and number of localizations per particle. Successful super-particle reconstruction is possible below a registration error of 25 nm. **d** Registration error of simulated PAINT data with 50% DOL and tilt angle of 60 degrees at different number of particles per dataset. **e** Registration error of simulated PAINT data with 75% DOL and arbitrary pose at different number of particles per dataset. Solid lines indicate the mean and shaded area show the standard error of the mean (*n* = 15).

The processing pipeline is built upon our previous 2D method^14^ with modifications to each step to handle 3D localizations (Fig. 1a) and with the addition of computational blocks for symmetry detection and, optionally, for symmetry promotion. Briefly, we first register all *N* segmented particles in pairs, which provides *N*(*N*−1)/2 relative registration parameters *M*_*ij*_ (3D rotation and translation from particle *i* to *j*). To find the absolute poses *M*_*i*_, we map the relative poses from the group of 3D rotations and translations, SE(3), to its associated Lie-algebra and then average them using an *L*_1_ norm for superior robustness (see “Methods” section)^15^. With the absolute poses, we then recompute the relative transformations to perform a consistency check for removing outlier and erroneous registration entries in the all-to-all matrix. These transformations are used to generate a data-driven template. Each single particle is then registered to density-resampled versions of this template for 3–5 iterations. During this process, prior knowledge of symmetry can be incorporated (see “Methods” section). The consistency check step also reveals useful information about the particle symmetry which can immediately be used to either promote symmetry in the iterated Lie-algebra averaging step or independently be used for quantitative structural analysis of the macromolecular complexes (see “Methods” section). We also propose a computationally efficient means of sorting and removing outlier particles (see “Methods” section). Finally, we needed to adapt our earlier 2D pipeline^14^, as 3D localization microscopy data typically has anisotropic localization uncertainty, being 2–4 times worse in the axial direction. In the initial step, however, an isotropic Gaussian Mixture Model (GMM) with multiple initializations regularly sampled on the rotation group is used to register the particles (see “Methods” section), and the best of these registrations is picked based on the Bhattachraya cost function that takes the anisotropic localization uncertainties into account. In case of large anisotropies the GMM registration possibly returns a sub-optimal registration preventing to reach the globally optimal solution. We evaluated this potential problem but found that after dense sampling of the initial GMM starting parameters in the rotation group (see “Methods” section) this did not play a role for our data.

### Particle fusion of 3D simulated SMLM data

We evaluated our algorithm using simulations of the Nup107 subcomplex of the NPC (Fig. 1c–e). Nup107 is a nucleoporin which is part of the Nup107-160 complex^16^, together with eight other nucleoporins. Our ground-truth model consists of 2 × 16 copies of Nup107 arranged in eight pairs on the two rings of the NPC, with a 13° azimuthal shift (Fig. 1b). The quality of registration was assessed with an error measure based on the residual registration error of the underlying binding sites (see “Methods” section), which is independent of the localization precision. We found that for registration errors smaller than the distance between the 8-fold symmetric subunits of the NPC rings (~25 nm) the reconstruction was sufficiently good that we considered the alignment to be a success (Supplementary Fig. 1).

We simulated both PAINT and STORM imaging, to assess how the switching kinetics of the fluorescent labels affects the particle fusion (Supplementary Fig. 2). For PAINT, we generated particles with a DOL of 75%, 50%, and 30%, localization uncertainties of 3, 8, and 13 nm in-plane and three times worse in the axial direction, and tilt angles spanning a range of ±36° (Supplementary Fig. 3). For STORM, we kept the DOL fixed at a realistic value of 50% while varying the average number of localizations per particle from 20 to 150 (corresponding to different fluorophore bleaching rates), and with the same range of localization uncertainties and tilt angles as before. For each simulation condition, we generated 15 datasets containing 100 particles each. We found that a registration error below 8–10 nm was required (Supplementary Fig. 1) to fully resolve the sixteen pairs of Nup107 sites. For PAINT, this was achieved for a minimum DOL of 50% and a localization precision better than 8 nm (Fig. 1c). For STORM, we observe that for high localization precision (~4 nm) the registration error is below 10 nm even for a low number of localizations per particle (down to 20). For a lower average localization precision of ~13 nm, the registration errors of all simulated STORM datasets were above 20 nm. This is similar to the error range of PAINT data at 30% DOL. Consistent with our previous work^14^, we observe that STORM data requires a higher DOL than PAINT to achieve a similar performance. The simulations also indicate that a high-quality reconstruction (error <10 nm) requires at least 50–100 particles (Fig. 1d) for PAINT data with 50% DOL. Even for unconstrained random pose variations and 75% DOL, the required number of particles for a successful registration remains relatively constant (Fig. 1e).

In Supplementary Fig. 4, we investigated if our pipeline is susceptible to potential symmetry of the underlying structure. We simulated three different particles (with arbitrary pose variation) of a highly symmetric (dodecahedron), a semi-symmetric (“building”), and a totally asymmetric structure (“ring-square”) and observed that our pipeline works properly for all these structures (see “Methods” section for simulation settings).

### Particle fusion of 3D experimental SMLM data

**Fig. 2:**
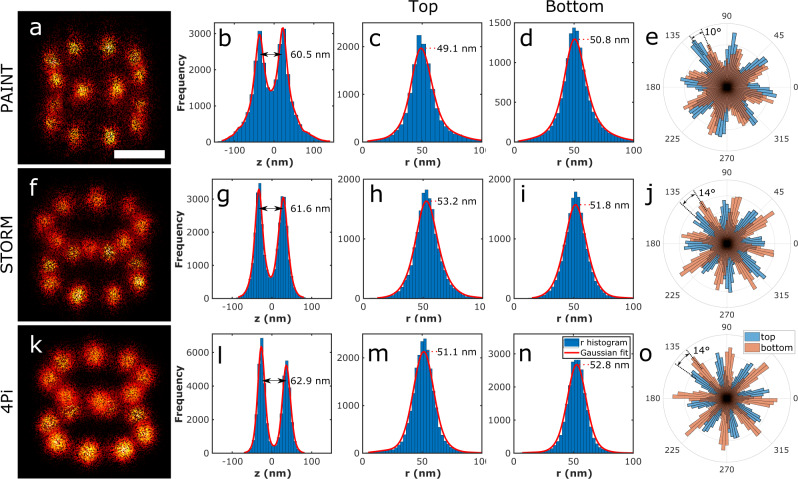
3D Particle fusion of Nup107 acquired with different 3D localization microscopy techniques. **a** Fusion of 306 particles acquire by 3D astigmatic PAINT. **b** Histogram of the Z coordinate of localizations in the super-particle. **c** Histogram of the radius of cytoplasmic ring localizations, **d** nuclear ring. **e** Rose plot of the localization distribution over azimuthal angles for the cytoplasmic (blue) and nuclear (orange) rings of the super-particle. **f** Fusion of 356 particles acquired by 3D astigmatic STORM. **g**–**j** Similar to **b–e**. **k** Fusion of 750 particles acquired by 4pi STORM. **l–o** Similar to **b–e**. Scale bar is 50 nm.

We applied our algorithm to experimental 3D SMLM images of NPCs in fixed U2OS cells (Fig. 2 and Supplementary Movies 1 and 2). Cells expressing Nup107-SNAP labeled with Alexa Fluor 647-benzylguanine were imaged with three different SMLM techniques, 3D astigmatic PAINT (Supplementary Figure 5-6), 3D astigmatic STORM^17,18^ and 4Pi STORM^19,20^. Figure 2a, f and k show the results of fusing 306, 356, and 750 manually segmented particles for the three modalities, which had an average number of localizations per particle of 88, 115 and 58, respectively. The final FSC resolution was ~15 nm (isotropic, see Supplementary Fig. 7). We measured the distance between the cytoplasmic and nuclear rings as 60.5, 61.6 and 62.9 nm for PAINT, STORM and 4Pi STORM data, respectively (Fig. 2b, g and l), and we determined the average radius to be 49.1, 53.2 and 51.1 nm for the top and 50.8, 51.8 and 52.8 nm for the bottom rings (Fig. 2c, d, h–i and m–n). Finally, the phase shift differences between the two rings (for analysis see ‘Methods’) were found to be 10°, 14° and 14° (Fig. 2e, j and o, and Supplementary Fig. 8). These measurements are in good accordance with cryo-EM-based models derived from the work of von Appen et al.^21^, who found a phase shift of 14°, height of 59 nm, outer ring radius of 49.7 nm, and inner ring radius of 46.6 nm. The experiments for NPCs in the lower nuclear membrane indicate a narrow tilt angle distribution (~12°, see Supplementary Fig. 9), well within the tilt tolerance limit assessed from the simulations.

### Macromolecular symmetry detection

**Fig. 3:**
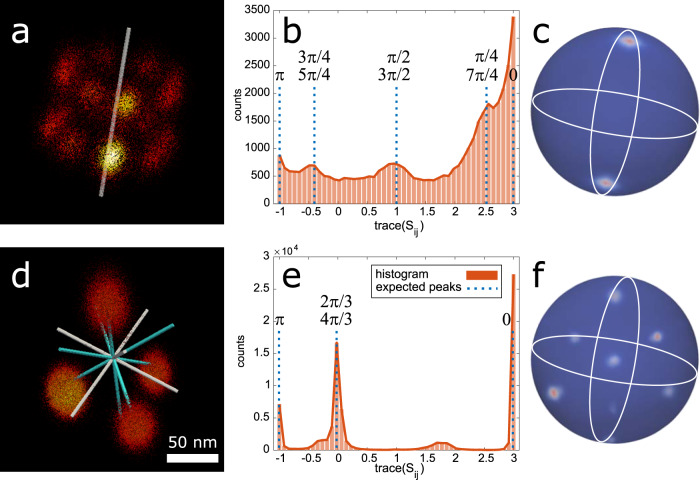
Symmetry group detection from SMLM data. **a** Fusion of 300 STORM Nup96 particles and the estimated rotational symmetry axis. **b** Histogram of the trace(*S*_*ij*_) reveals the 8-fold rotational symmetry of the Nup96 protein. **c** Density plot of the estimated axes of rotation on the unit sphere for the Nup96 dataset. **d** Fusion of 400 3D PAINT DNA-origami tetrahedron particles and the estimated axes of rotation. The white and cyan bars indicate the 3-fold and 2-fold rotational symmetry axes, respectively. **e** Histogram of the trace(*S*_*ij*_) reveals the 3- and 2-fold rotational symmetries of the tetrahedron structure. **f** Density plot of the estimated axes of rotation on the unit sphere. The dense regions on the unit sphere in **c**, **f** project the orientation of the estimated axis(es) of rotations.

In Fig. 3 and Supplementary Movies 3 and 4, we depict our symmetry group detection approach for experimental STORM images of Nup96^16^ (https://www.ebi.ac.uk/biostudies/BioImages/studies/S-BIAD8) and for experimental tetrahedron DNA-origami PAINT images. Figure 3a, b show the result of fusing 300 NPC particles after Lie-algebraic averaging (before the final bootstrapping step) together with the estimated symmetry group and its axis of rotation. The deviation from the unit matrix of $S_{ij} = R_{ij}( {\hat R_{ij}} )^{ - 1}$, the mismatch between the initially estimated rotation between a particle pair (*R*_*ij*_) and the inverse of the one after Lie-algebraic averaging ($\hat R_{ij}$), carries information about the symmetry of the particles (see Fig. 1a and “Methods” section). The peaks of the experimental histogram of trace(*S*_*ij*_) for this dataset are located at $\left\{ {3,1 + \sqrt 2 ,1,1 - \sqrt 2 , - 1} \right\}$ which correspond to $\left\{ {1 + 2\cos \left( {2\pi k/n} \right)|k = 0,1, \ldots ,n - 1} \right\}$ with *n* = 8, providing quantitative empirical evidence for the 8-fold rotational symmetry. As for the tetrahedron, we fused 256 tetrahedron-shaped DNA-origami nanostructures acquired with PAINT (Supplementary Figs. 10–12). A tetrahedron has a 2- and 3-fold rotational symmetry with seven independent axes of rotation. The experimental histogram of trace(*S*_*ij*_) shows significant peaks at three locations which are the union of the sets {3, − 1} and {3, 0} for 2 and 3-fold rotational symmetry (Fig. 3d, e). For both structures, the orientation of the symmetry axes are determined from the data as well, by localizing the maxima in the density plot of the rotation axes of the *S*_*ij*_ matrices (see Fig. 3c, f and “Methods” section).

## Discussion

We have developed a general purpose, template-free 3D particle fusion algorithm for SMLM that is robust to typical experimental conditions, and have shown its performance on simulated data, on the Nup96 and Nup107 subcomplexes of the NPC for three different imaging setups, and on DNA-origami tetrahedrons. By increasing the effective DOL and improving the SNR, our pipeline opens up possibilities for reliable identification of protein locations within macromolecular complexes, thereby adding specificity to EM-SPA methods via correlative approaches. We show that as few as 50 particles are enough for this purpose, enabling the exciting possibility to detect transient, rarely populated states. In addition, we provide an efficient computational approach for detecting structural symmetry from the image data, with access to the rotational multiplicity and the rotational axes.

## Methods

### Nup107 sample preparation for astigmatic PAINT

U2-OS cells were passaged every other day and used between passage number 5 and 20. The cells were maintained in DMEM supplemented with 10% Fetal Bovine Serum and 1% Penicillin/Streptomycin. Passaging was performed using 1× PBS and Trypsin-EDTA 0.05%. In all, 24 h before immunostaining, cells were seeded on ibidi eight-well glass coverslips at 30,000 cells/well.

Prefixation was performed with prewarmed 2.4% Paraformaldehyde (PFA) for 20 s followed by the permeabilization at 0.4% Trion-X 100 for 10 s. Next, cells were fixed (main fixation) with 2.4% PFA for 30 min. After 3× rinsing with 1× PBS the cells were quenched with 50 mM Ammoniumchloride (in 1× PBS) for 4 min. Then, cells were washed 3× with 1×PBS followed by incubation in 1× PBS for 5 min twice. For SNAP-labeling, cells were incubated with 1 μM of SNAP-ligand-modified DNA oligomer in 0.5% BSA and 1 mM DTT for 2 h. Finally, cells were washed 3× for 5 min in 1× PBS, incubated with 1:1 dilution of 90 nm gold particles in 1× PBS as drift markers, washed 3 × 5 min and immediately imaged.

### Nup107 sample preparation for 4PI STORM

The U2OS cells were seeded on 18 mm #1.5 round coverslips which had been sterilized in 70% ethanol, dried and washed three times with 1x PBS. All coverslips used for 4Pi-SMLM were coated with a mirror-reflective aluminum film over one quarter of their surface, for the purpose of alignment in the 4Pi microscope. Mirror coating was accomplished using a thermal evaporator at the Optics Workshop of the Max-Planck-Institute for Biophysical Chemistry, Göttingen. Seeded cells were allowed to attach overnight at 37°C and 5% CO2 in a cell culture incubator.

Cells were rinsed twice with PBS and pre-fixed with 2,4% paraformaldehyde (PFA; Electron Microscopy Sciences; cat.# 15710) in PBS (+Ca^2+^/Mg^2+^) for 30 seconds. The cells were then immediately permeabilized with 0.5% Triton X-100 (Sigma-Aldrich; cat.# T8787) in PBS (+Ca^2+^/Mg^2+^) for 10 min and directly fixed afterwards with 2,4% paraformaldehyde (PFA; Electron Microscopy Sciences; cat.# 15710) in PBS (+Ca^2+^/Mg^2+^) for another 30 min. After fixation, the samples were rinsed three times with PBS and quenched for remaining fixative with 50 mM NH_4_Cl for 5 min. After quenching, the sample was rinsed three times with PBS and washed three times for 5 min. with PBS. The fixed samples were immediately stained using one of the protocols described below.

In order to perform NPC labeling with SNAP-tag and after fixation, samples were blocked with a few drops of Image-iT FX Signal Enhancer (Thermo-Fisher; cat.# I36933) for 30 min. The benzylguanine (BG)-conjugated AF647 (SNAP-Surface; NEB; cat.# S9136S) was diluted to 1 μM in blocking solution (0,5% (w/v) BSA, 1 mM DTT in 1x PBS) and incubated with the sample for 1 hour. This was followed by a final round of three rinsing and 5 min washing steps.

### DNA-origami tetrahedron sample preparation for PAINT

The tetrahedron DNA-origami structures were formed in a one-pot reaction with a 50 μl total volume containing 10 nM scaffold strand (p8064), 100 nM core staples, 100 nM connector staples, 100 nM vertex staples, 100 nM biotin handles, 100 nM DNA-PAINT handles, and 1400 nM biotin anti-handles in folding buffer (1× TE (5 mM Tris, 1 mM EDTA) buffer with 10 mM MgCl_2_). The solution was annealed using a thermal ramp cooling from 80 to 4 °C over the course of 15 h. After self-assembly, the structures were mixed with 1× loading dye and then purified by agarose gel electrophoresis (1.5% agarose, 0.5× TAE, 10 mM MgCl_2_, 1× SYBR Safe) at 3 V/cm for 3 h. Gel bands were cut, crushed, and filled into a Freeze ‘N Squeeze column and spun for 5 min at 1000 × *g* at 4 °C.

### Nup107 sample preparation for astigmatic STORM

The procedure followed is equal to the one described in Li et al.^18^. For convenience here the procedure is described also. Rinse 2x Coverslips containing Nup96-SNAP-tag cells (catalog no. 300444,CLS Cell Line Service) with warm PBS. In a 2.4% (w/v) formaldehyde(FA) in PBS solution for 40 s we preform prefixation before the samples were permeabilized in 0.4% (v/v)Triton X-100 in PBS for 3 min. Complete fixation was carried out in 2.4% (w/v) FA in PBS for 30 min followed by 3 Å~ 5 min washing steps in PBS after fixation. Quenching of FA was done by placing the samples in 100 mM of NH4Cl in PBS for 5 min and afterward washed 3x in PBS for 5 min each. Then, the sample was incubated for 30 min with Image-iT FX Signal Enhancer (catalog no. I36933, Thermo Fisher Scientific) and then stained with SNAP dye buffer (3 μM BG-AF647 (catalog no. S9136S, New England Biolabs) and 3 μM dithiothreitol in 0.5% (w/v) bovine serum albumin (BSA) in PBS) for 2 h at room temperature. We removed unbound dye by washing the coverslips 3x for 5 min in PBS. Samples were then mounted into custom sample holders in imaging buffers (50 mM of Tris/HCl pH 8, 10 mM of NaCl, 10% (w/v) d-glucose, 500 μg ml^−1^ of glucose oxidase, 40 μg ml^−1^ of glucosecatalase and 35 mM of MEA in H_2_O). We sealed the holder with parafilm.

### Single molecule experiments for astigmatic PAINT imaging of Nup107

Fluorescence imaging was carried on an inverted microscope (Nikon Instruments, Eclipse Ti2) with the Perfect Focus System, applying an objective-type TIRF configuration with an oil-immersion objective (Nikon Instruments, Apo SR TIRF ×100, numerical aperture 1.49, Oil). A 561-nm (MPB Communications Inc., 2W, DPSS- system) laser was used for excitation. The laser beam was passed through cleanup filters (Chroma Technology, ZET561/10) and coupled into the microscope objective using a beam splitter (Chroma Technology, ZT561rdc). Fluorescence light was spectrally filtered with an emission filter (Chroma Technology, ET600/50 m and ET575lp) and imaged on a sCMOS camera (Andor, Zyla 4.2 Plus) without further magnification, resulting in an effective pixel size of 130 nm (after 2 × 2 binning).

Imaging was carried out using an imager strand concentration of 1 nM (P3-Cy3B) in cell imaging buffer (buffer C) 30,000 frames were acquired at 200 ms integration time. The readout bandwidth was set to 200 MHz. Laser power (@561 nm) was set to 130 mW (measured before the back focal plane (BFP) of the objective), corresponding to 0.73 kW/cm^2^ at the sample plane.

Axial calibration was presented earlier in Li et al.^17^. Here the procedure is repeated for convenience. We dilute Tetra-Speck beads (0.75 μl from stock, catalog no. T7279, Thermo Fisher in 360 μl H_2_O, mixed with 40 μl 1 M MgCl_2_ and then put them on a coverslip in a custom-manufactured sample holder. After 10 min, the mix was replaced with 400 μl H_2_O. About 20 positions on the coverslip were defined with the use of Micro-Manager and the beads were imaged in z stacks (−1 to 1 μm, 10-nm step size) using the same filters as used in the intended experiment.

### Single molecule experiments for astigmatic PAINT imaging of Tetrahedron

Tetrahedron imaging experiments were carried out on an inverted Nikon Eclipse Ti microscope (Nikon Instruments) with the Perfect Focus System, attached to a Yokogawa spinning disk unit (CSU-W1, Yokogawa Electric). An oil-immersion objective (Plan Apo ×100, NA 1.45, oil) was used for all experiments. The excitation laser (561 nm, 300 mW nominal, coherent sapphire or 532 nm, 400 mW nominal, Cobolt Samba) was directly coupled into the Yokogawa W1 unit using a lens (focal length *f* = 150 mm). The pinhole size of the disk was 50 μm. As dichroic mirror, a Di01-T405/488/568/647-13 × 15 × 0.5 from Semrock or t540spxxr-uf1 from Chroma was used. Fluorescence light was spectrally filtered with emission filters (607/36 nm from Semrock or ET585/65 m + ET542lp from Chroma) and imaged on an EMCCD camera (iXon 897, Andor Technologies), resulting in a pixel size of 160 nm. The power at the objective was measured to be ~10% of the input power.

For the tetrahedron imaging experiment (2 nM of P1-Cy3b imager in buffer B) the Andor iXon 897 with a readout bandwidth of 5 MHz at 16 bit and 5× pre-amp gain was used. The EM gain was set to 100. In all, 30,000 frames with an integration time of 800 ms were acquired. Imaging was performed using the Yokogawa W1 spinning disk unit with an excitation intensity of ~226 W/cm^2^ at 561 nm at the sample (laser was set to ~38 mW). No additional magnification lens was used resulting in an effective pixel size of 160 nm.

3D images were acquired using a plan-convex cylindrical lens with a focal length of *f*  = 0.5 m, ~2 cm away from the camera chip. The calibration was done as in earlier studies. For the processing of the data the software package Picasso^22^ was used.

### Single molecule experiments for astigmatic STORM imaging of Nup107

homozygous Nup107-SNAP U2-OS cell lines were fixed and labeled with Alexa Fluor 647-benzylguanine and imaged on a custom-built setup that contains a cylindrical lens in the emission path for astigmatic 3D localization. The data were fitted using an experimental PSF model calibrated using a z-stack of beads that were immobilized on the coverslip^17^. Subsequently, fitting errors induced by the refractive index mismatch were corrected based on a calibration of beads immobilized in a gel^18^. See Li et al.^18^. for additional description.

### Single molecule experiments for 4Pi STORM imaging of Nup107

The design of the 4Pi microscope was based on an earlier design published by Aquino et al.^19^, which was then extensively modified to achieve higher image quality and usability. Specifically, the design was changed to incorporate feedback systems to maintain the sample focus position, higher NA objectives to collect more light, a completely redesigned sample stage allowing for fast and reliable sample mounting and linear translation when adjusting the sample position, a redesigned 4Pi image cavity allowing for maintenance of the beam path alignment, and new acquisition and control software to allow accurate control of the instruments involved in the system stabilization and acquisition of the raw image data. The laser illumination sources used for STORM imaging included a red laser for imaging (642nm CW, 2W, MPB Communications Inc.) and a UV laser for molecule re-activation (405nm CW, 100mW, Coherent). Excitation light was controlled and modulated either directly via the laser controller or via an acousto-optic tunable filter (AA Opto Electronic). Variable angle TIRF or near-TIRF illumination was achieved by coupling all light sources through an optical fiber, whose output was positioned in an optical plane conjugate to the objective lens back focal plane. By placing the output of the fiber on a motorized translation stage, the illumination angle could be continuously varied for optimal signal to background ratio. The 4Pi microscope cavity was based on two high-NA objective lenses (Olympus, 100x, silicone oil immersion, NA 1.35). One objective was fixed in position on a mounting block while the other was adjustable in three dimensions using a 3-axis piezo stage (Physik Instrumente, P-733.3). The adjustable objective was also adjustable in tip/tilt and XYZ via micrometer screws for coarse positioning and alignment. Illumination and control beams were introduced into the 4Pi cavity and brought out again via dichroic mirrors (ZT405-488-561-640-950RPC, Chroma). The detected fluorescence from the two objectives was recombined at a 50:50 beam-splitter (Halle). Prior to the beam-splitter each detected beam passed through a quarter wave plate (Halle) and a custom Babinet-Soleil compensator made of quartz and BK7 glass, one of which with an adjustable thickness of quartz glass, which allowed a precise phase delay to be introduced between the P- and S- polarized fluorescence light. The remainder of the detection path consisted of an optical relay to crop and focus the overlaid P- and S- polarized images onto four quadrants of an EMCCD camera (Andor Ixon DU897) as previously described. Before the camera, the light was filtered with fluorescence emission filters (Semrock LP647RU, Semrock FF01-770SP) and optionally a dichroic mirror (Semrock FF685-Di02) which allowed the fluorescence in one polarization channel to be filtered selectively for two-color 4Pi-SMLM imaging. Control systems included the sample focus control and the objective alignment control, and each of these was based on an infra-red laser beam introduced into the 4Pi cavity. The sample focus control was based on a design similar to that used in a standard STORM microscope: an infrared beam (830nm laser diode, Thorlabs) was reflected from the sample-glass interface, and the position of the reflected beam was detected on a photodetector. Fine control of the sample position was maintained with a linear piezo stage (Physik Instrumente, P-752) mounted underneath the top section of the three-axis linear stage used for sample positioning (Newport, M-462-XYZ-M). For the objective alignment control, a second infra-red beam (940nm laser diode, Thorlabs) was collimated and passed through the two objective lenses, focusing at the sample plane. Any motion of the two objectives with respect to each other resulted in a lateral shift in the transmitted beam, or a change in the collimation of the transmitted beam. The lateral shift was continuously monitored via a quadrant photodiode, and the transmitted beam collimation was monitored by splitting the beam and focusing it onto two pinholes positioned on either side of the focus, with photodetectors behind each pinhole. These signals were measured using a DAQ card (National Instruments), and a software-based feedback loop was then used to adjust the position of the movable objective lens to keep it aligned with the fixed objective lens. All microscope control and data acquisition were performed using custom software written in Labview (National Instruments).

The sample was illuminated with 642 nm excitation light in order to switch off the fluorophores and cause them to blink stochastically. The emitted light was filtered spectrally (see above) and detected at the EMCCD camera, running at a frame rate of 101 Hz. Typically, 100000 image frames were acquired in a single measurement. During the experiment, the power of the 405 nm laser was manually adjusted to re-activate the fluorophores and keep the number of localizations per frame constant. Optical stabilization of the z-focus (focus-lock) was engaged before starting each recording, in order to minimize sample drift during the measurement. Prior to each set of 4Pi measurements, images of a fluorescent bead located on the sample were recorded as the bead was scanned in the Z-dimension, in order to create a calibration scan which was used in post-processing analysis of the 4Pi STORM image data. For all experiments, images of beads located at different positions in the sample plane were recorded, in order to generate a coordinate mapping which allowed the coordinate systems of the different image channels to be mapped onto each other.

STORM image analysis and reconstruction follows a standard approach based on peak finding and localization^23^. Correction of sample drift in post-processing was done based on image correlation of the 3D STORM data with itself over multiple time windows. STORM images were rendered as summed Gaussian peaks with a Gaussian width approximately equal to the previously measured localization precision (typically 3.5 nm in X, Y, and Z).

### Data fusion pipeline

Our data fusion framework is largely the same as our earlier work^14^ with 3D instead of 2D localization data and with significant modification and improvement of each step. We equipped the Gaussian Mixture Model (GMM) registration with a routine for automatic (isotropic) scale selection which eliminate the need for parameter tuning (Supplementary Note 1 and Supplementary Fig. 13). In general, we found that the scale seems to be dependent on the shape of the particle rather than the DOL and localization precision and it may be recommended to fine-tune this scale parameter using this routine for the reconstruction of new types of particles. We further modified the initialization step of the GMM registration in order to uniformly cover the whole SO(3) landscape for the initial pose of each particle (Supplementary Note 2) and increased the sampling. As the GMM does not convergence to a global optimal solution we evaluate all found registration parameters with the Bhattachraya cost function, to select the optimal registration parameters. In this way we can also take the anisotropic localization precision in 3D into account. In case of large anisotropic localization uncertainties in all dimensions the GMM registration might return the wrong registration and we might not find the globally optimal solution by this procedure. The registration parameters could, however, further be refined by optimizing with the Bhattachraya cost starting from the best GMM registration in this case. We tried this but found that it did not improve the result a lot while being computationally demanding.

In order to find the absolute pose of the particles from the relative pairwise transformations, we used Lie-algebraic averaging as described in ref. ^14^. Here, we used the *L*_1_ norm which has significantly better performance than the *L*_2_ norm in the presence of outlier and erroneous registrations (see Supplementary Note 3 and Supplementary Figs. 14 and 15 for a detailed comparison). We have to replace the consistency evaluation as rotations in 2D can be characterized by one in-plane angle only and therefore a straightforward threshold can be applied to the angle difference. In 3D, the three Eulerian angles are required to describe a rotation which complicates matters significantly as different rotations do not commute. To this end we make use of the fact that the recomputed relative transforms $\hat R_{ij}$ (rotation) should ideally match the initially measured relative transformations *R*_*ij*_, i.e., in the ideal case

1 $$S_{ij} = R_{ij}( {\hat R_{ij}} )^{ - 1} = I$$

in which *I* is the 3 × 3 identity matrix with trace(*S*_*ij*_) = 3. In practice, and due to the registration error, trace(*S*_*ij*_) can be <3. Therefore, a reasonable choice for the consistency check is to remove the transformations that deviate from the peak at 3 more than a certain threshold (*∈*). Here, we set an empirical threshold *∈* = 0.5 to remove inconsistencies.

### Symmetry detection

Registration errors are not the only reason that might lead to a deviation of *S*_*ij*_ from identity. In fact, trace(*S*_*ij*_) can no longer be assumed to be close to 3 in case of a rotational symmetry. Any found $\hat R_{ij}$ that adheres to the symmetry constraint is a valid solution to the registration problem and therefore the *S*_*ij*_ are expected to be close to any of the transformations $\bar S_k$ in the symmetry group (labeled with index *k*). The numerically found solution *S*_*k*_ appears to be random, which enables us to experimentally assess the symmetry. If, for example, the symmetry group contains an *n*-fold rotational symmetry, then the $\bar S_k$ could be an *n*-fold rotation matrix. The trace of the rotation matrices can be expressed as^24^:

2 $${\mathrm{trace}}( {S_{ij}} ) = 1 + 2\cos \psi _{ij},$$

where *ψ*_*ij*_ are the rotation angles. If we plot the histogram of the found trace(*S*_*ij*_), we expect peaks at values 1 + 2cos(2*πk*/*n*), for *k* = 0, 1, …, *n*−1. It should be mentioned that these peaks are typically spread due to the aforementioned registration error. For example, for a 2-fold rotational symmetry we have peaks at {3,−1}, for a 3-fold rotational symmetry at {3,0} and for an 8-fold rotational symmetry at $\left\{ {3,1 + \sqrt 2 ,1,1 - \sqrt 2 , - 1} \right\}$.

Further, we can also compute the axes of rotation from *S*_*ij*_ using the following formula:

3 $$u_{ij} = \left( {\begin{array}{*{20}{c}} {s_{ij}^{3,2} - s_{ij}^{2,3}} \\ {s_{ij}^{1,3} - s_{ij}^{3,1}} \\ {s_{ij}^{2,1} - s_{ij}^{1,2}} \end{array}} \right)$$

in which $s_{ij}^{m,n}$ are the elements of the 3 × 3 *S*_*ij*_ matrix. This will provide us with *N*(*N*−1)/2 estimated rotation axes. To infer the symmetry axis(es) of the particle, each estimated axis is normalized to have unit length and then is projected as a point on the unit sphere. The maxima in the density plot of these points reveals the symmetry axes (Fig. 3c, f).

Finally, it turned out that iterating the loop of Lie-algebraic averaging based on the most consistent *S*_*ij*_ (those for which trace(*S*_*ij*_) ≈ 3) and the re-computation of the relative transformations can better reveal an indication of symmetry in the trace histogram. This can be considered as an extension of the outlier removal step.

### Symmetry promotion during Lie-algebra averaging

In step 2 of the pipeline, we recompute the relative transformations using the Lie-algebra averaging of the consistent transformations for which trace(*S*_*ij*_) ≈ 3. This hard thresholding, however, discards the transformations that are multiples of the rotational symmetry group(s) of the underlying structure and are located at 1 + 2cos*ψ*_*ij*_ with *ψ*_*ij*_ = 2*πk*/*n* for *n*-fold symmetry group. In this extra step of the pipeline, we can use the symmetry group information (either found from the symmetry detection step or obtained from initial prior knowledge) in the Lie algebra averaging to transform the *S*_*ij*_ with the retrieved symmetry transformation $\bar S_k$ to another transformation $\bar S_{ij} = S_{ij}\left( {\bar S_k} \right)^{ - 1}$, that is close to unity, so that the principles of Lie-algebra averaging can again be applied. This increases the number of consistent transformations, allows for a more robust Lie-algebra averaging and subsequently a better data-driven template for the bootstrapping step (Supplementary Fig. 16 and Supplementary Movie 5). The increase of the number of consistent registrations is foremost dependent on the initial quality of registrations and the multiplicity of the symmetry present.

### Symmetry promotion after bootstrapping

For symmetric structures and in the case of underlabeling or of a non-uniform distribution of localizations per binding sites (e.g. in STORM), the hotspot problem reported earlier^14^ is unavoidable. The registration algorithm tends to match dense regions of the structure and consequently the unbalanced occupancy of sites is reinforced in the process. We overcome this problem by properly incorporating prior knowledge about the symmetry group of the structure. For NPC, which has an 8-fold rotational symmetry (2D cyclic group *C*_8_) around the estimated rotation axis through the center of the cytoplasmic and nuclear rings, we randomly added integer multiples of 2*π*/8 to the alignment angles of the particles at each iteration of the bootstrapping. This subsequently results in a uniform distribution of localizations over the binding sites. It is worth mentioning that this approach is different from what is done in single particle averaging in EM^25^ and in the method of Sieben et al.^11^, where the asymmetrical subunit of the particles is replicated to generate a symmetric structure based on the given symmetry group. In our approach the final reconstruction is mathematically not symmetric, but the symmetry is used to resolve the hotspot problem. This approach can easily be adapted to other simple point groups such as cyclic *C*_*n*_ and dihedral *D*_*n*_ groups given the axis (or axes) of rotation(s).

### Outlier particle removal

In our earlier work^14^, we kept all initially picked particles for the final super-particle. We only removed bad registrations from the all-to-all matrix, keeping the graph connected. In practice, however, it happens that the segmented particle set contains “outliers” that are either not a particle but background or just very low-quality particles. We propose a simple and efficient method for excluding outliers with small computational cost. After the bootstrapping step, we construct an *N* × *N* matrix with elements equal to the Bhattacharya cost function for all pairs of aligned particles (Supplementary Fig. 17a). We sum over the columns (or rows) of this similarity matrix to assign a single score to each individual particle. If all particles are of good quality, these scores should be similar in magnitude. For outlier particles, however, we observe that the histogram of scores has an extended tail. We therefore identify outliers as particles with scores that are more than three scaled median absolute deviations (MAD) away from the median (Supplementary Fig. 17b). This outlier particle removal only works properly if most of the segmented particles are of good quality and the particle fusion has not failed. The visual experience of the final reconstruction is barely affected for the examples shown in Fig. 2, however, the best and worst particles demonstrate how this approach can rank the quality of the included particles (Supplementary Fig. 17c, d).

### Simulation setup

Our first ground-truth model consists of 2 × 16 copies of Nup107 arranged in eight pairs on the cytoplasmic and nuclear ring of the NPC with ~13° of azimuthal shift (Fig. 1b). PAINT and STORM switching kinetics were simulated as earlier described^14^. For each parameter setting, we generated 15 datasets containing 100 particles each. The ground-truth model for dodecahedron has 20 binding sites at its vertices with a minimum binding site distance of 30.9 nm (Supplementary Fig. 4a, b). The “building” model has also 20 binding sites forming a structure with dimensions of 20 × 100 × 60 (width × length × height) nm (Supplementary Fig. 4c, d). The “ring-square” structure consists of an unconnected 8-point square and an 8-point ring. The center of the ring has an offset with respect to the center of the square and the ring is tilted, making a 26.6° angle with the horizontal plane in such a way as to break any possible symmetry (Supplementary Fig. 4e, f). We used these models to simulate PAINT particles with a DOL of 50 and 75% and a photon count of 2000 and 5000.

### Registration error measure in simulations

To assess the performance of the method on simulated data, we devised an error metric which is independent of the shape of the ground-truth super-particle, does not have a global offset problem i.e. any transformation of the whole ensemble of particles gives the same error, can solve the symmetry ambiguity, is not impaired by underlabeling, and has the same physical unit as the localization data. The error is the averaged Euclidean distance between corresponding binding sites after applying the data fusion process. This works in simulation only as there we know the ground truth and thus, we can establish point correspondence between binding sites. This measures the registration error, however, if we would do the same with the localization data, we would get a convoluted compound of registration error and localization error and an overweighting of sites with many localizations. In Supplementary Figs. 18 and 19, we illustrate the process. We find the point correspondence by measuring the distance for all possible combinations of binding sites and then report the minimum as the registration error between the two particles. Supplementary Figure 19 demonstrates such combinations for a simplified NUP structure with *K* = 16 designed binding sites. Mathematically, the registration error of *N* aligned particles is computed as follows:

4 $${\mathrm{error}} = \frac{2}{{N\left( {N - 1} \right)}}\left( \mathop{\sum}\limits_{m = 1}^{N - 1} \mathop{\sum}\limits_{n = m + 1}^{N} \mathop {{\min }}\limits_{i - 1, \ldots ,K} \sqrt {\frac{1}{K}\mathop {\sum}\nolimits_{j = 1}^K \left\Vert{x_m\left( j \right) - x_n\left( {{\mathrm{mod}}\left( {i + j,K} \right)} \right)}\right\Vert ^2}\right)$$

in which *x*_*m*_ is the localization data (3D coordinate) of particle *m* from the set of all particles. The double summation is over all pairs of particles and over all possible correspondences of the binding sites for the current pair of particles.

### Analysis of NPC structural parameters

NPCs are embedded in the nuclear membrane and their tilt axis aligns reasonably with the optical axis (normal distribution with about zero mean, Supplementary Fig. 9). Consequently, the Lie-algebra always aligns the particles with the *xy* plane for experimental data. A moment analysis of the super-particle is used to align the average pose with the principle planes (*xy*, *xz*, *yz* and etc.), i.e., aligning the symmetry axis of the NPC super-particle with the *z*-axis. The distance between the upper and lower rings of the NPCs is estimated by first computing the histogram of the *z* coordinate of the localization data in the super-particle. Then, a kernel-smoothing distribution with a bandwidth of 4 nm is fitted to the histogram and, finally, the distance between the two peaks of the fit is computed (Fig. 2b, g and l). The radius of the two rings is measured by separating the localization data of the super-particle in two halves using a segmentation threshold which is computed as the local minimum of the *z* coordinate histogram. Then, the *x* and *y* coordinates of the localization data are transformed to two-dimensional polar coordinates (*r*, *θ*). The peak of the histogram of the *r* component of the localizations defines the radius of the rings (Fig. 2c–d, h–i and m–n). The angular shift between the two rings of the Nup107 is estimated by first fitting the function *b*_0_ + *b*_1_ sin(8*θ* + *b*_2_) to the angular components of the localization data in each ring. The iterative least squares method is used for this nonlinear regression model to find the unknown coefficients *b*_0_, *b*_1_ and *b*_2_. Then, the difference between the fitted *b*_2_ parameters for the two rings defines the angular phase difference (Supplementary Fig. 8).

### Computational complexity

All-to-all registration of 306 and 356 Nup107 PAINT and STORM particles with an average number of localization per particle of 88 and 115 took about 1 and 2 h on a desktop PC (CPU: Intel® Xeon(R) Silver 4110 CPU @ 2.10 GHz × 32, RAM: 32 Gb and GPU: TITAN Xp).

### Reporting summary

Further information on research design is available in the Nature Research Reporting Summary linked to this article.

## Supplementary information

Supplementary Information

Description of Additional Supplementary Files

Peer Review File

Reporting Summary

Supplementatary Movie 1

Supplementatary Movie 2

Supplementatary Movie 3

Supplementatary Movie 4

Supplementatary Movie 5

## Data Availability

The authors declare that all other data supporting the findings of this study are available within the paper and its supplementary information files. Localization data for NUP107 and tetrahedron 3D DNA-origami data are available through 4TU Research Data repository^26^ at 10.4121/13797686. NUP96 localization data are available publicly at https://www.ebi.ac.uk/biostudies/BioImages/studies/S-BIAD8. Source data are provided with this paper.

## Source data

Source Data

## Code availability

The software is available for download under the terms of the Apache2.0 license from Github and 4TU Research Data repository^27^ at https://github.com/imphys/smlm_datafusion3d. (10.4121/13743703).
