# Publisher Correction: 3D particle averaging and detection of macromolecular symmetry in localization microscopy

**DOI:** 10.1038/s41467-021-23767-9

**Published:** 2021-05-26

**Authors:** Hamidreza Heydarian, Maarten Joosten, Adrian Przybylski, Florian Schueder, Ralf Jungmann, Ben van Werkhoven, Jan Keller-Findeisen, Jonas Ries, Sjoerd Stallinga, Mark Bates, Bernd Rieger

**Affiliations:** 1 Department of Imaging Physics, Delft University of Technology, Delft, The Netherlands; 2 Department of NanoBiophotonics, Max Planck Institute for Biophysical Chemistry, Göttingen, Germany; 3 Department of Physics and Center for Nanoscience, Ludwig Maximilian University, Munich, Germany; 4 Max Planck Institute of Biochemistry, Martinsried, Germany; 5 Netherlands eScience Center, Amsterdam, The Netherlands; 6 Cell Biology and Biophysics Unit, European Molecular Biology Laboratory (EMBL), Heidelberg, Germany

**Keywords:** Super-resolution microscopy, Structure determination

Correction to: *Nature Communications* 10.1038/s41467-021-22006-5, published online 14 May 2021.

The original HTML version of this Article was updated shortly after publication because the previous HTML version linked to an incorrect Supplementary Information file.

## Supplementary information

Supplementary Information

