# Supplementary material for: 3D particle averaging and detection of macromolecular symmetry in localization microscopy
Source: Nat Commun. 2021 May 14;12:2847. doi: 10.1038/s41467-021-22006-5 (PMC8121824; doi:10.1038/s41467-021-22006-5)
Supplement: Supplementary file 1 — Supplementary Information [file 41467_2021_22006_MOESM1_ESM.pdf]

Supplementary Information

3D particle averaging and detection of macromolecular symmetry in localization microscopy

Hamidreza Heydarian, Maarten Joosten, Adrian Przybylski, Florian Schueder, Ralf Jungmann, Ben van Werkhoven, Jan Keller-Findeisen, Jonas Ries, Sjoerd Stallinga, Mark Bates, Bernd Rieger

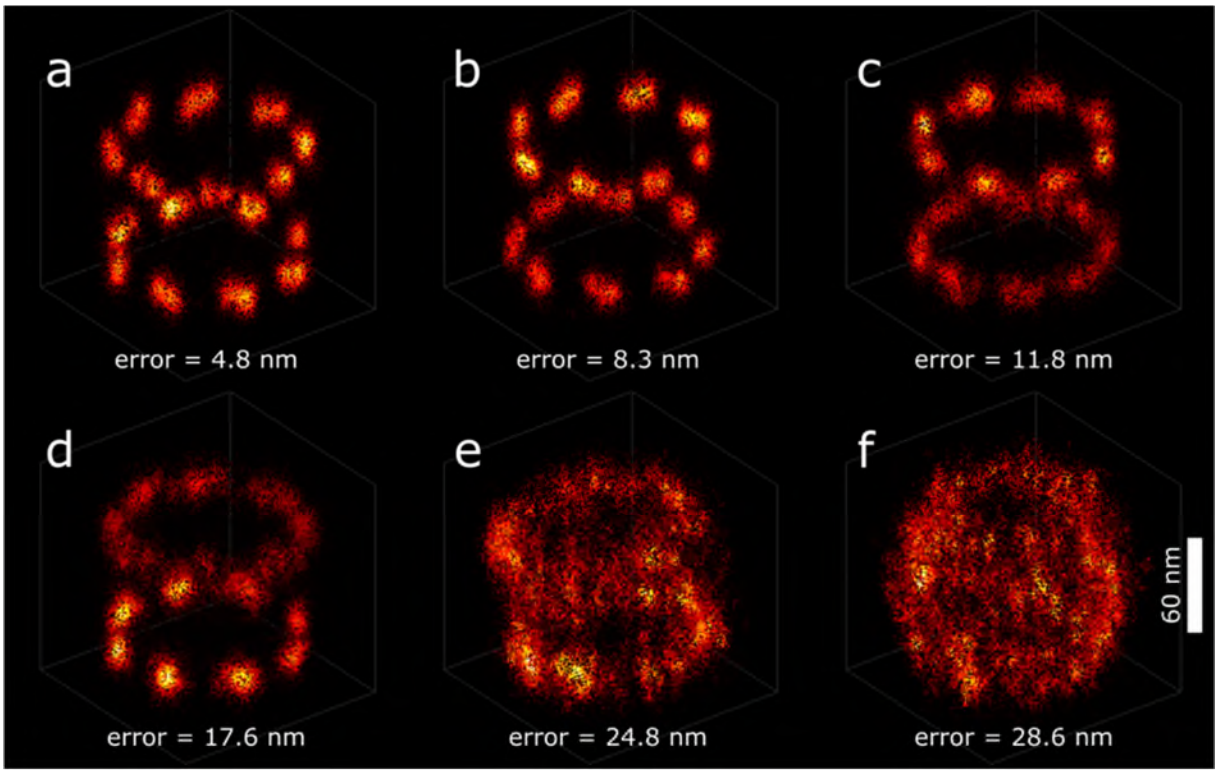

Supplementary Figure 1 | Visual appearance of the superparticle for different value of the proposed registration error metric. (a-f) Superparticles are the result of stacking 100 simulated STORM particles with an average localization uncertainty of 4 nm and 50% DOL. For each superparticle, ground-truth transformations are altered to make the group-wise alignment worse. From top to bottom and left to right, the measured error is increasing which also visually matches the quality of the reconstructions. For the error less than ~10 nm (a-b), the double blobs are still recognizable. For larger error as in (c-d), the double blobs merge into a single blob and for errors above ~20 nm the reconstructions lose the geometrical features of the ground-truth.

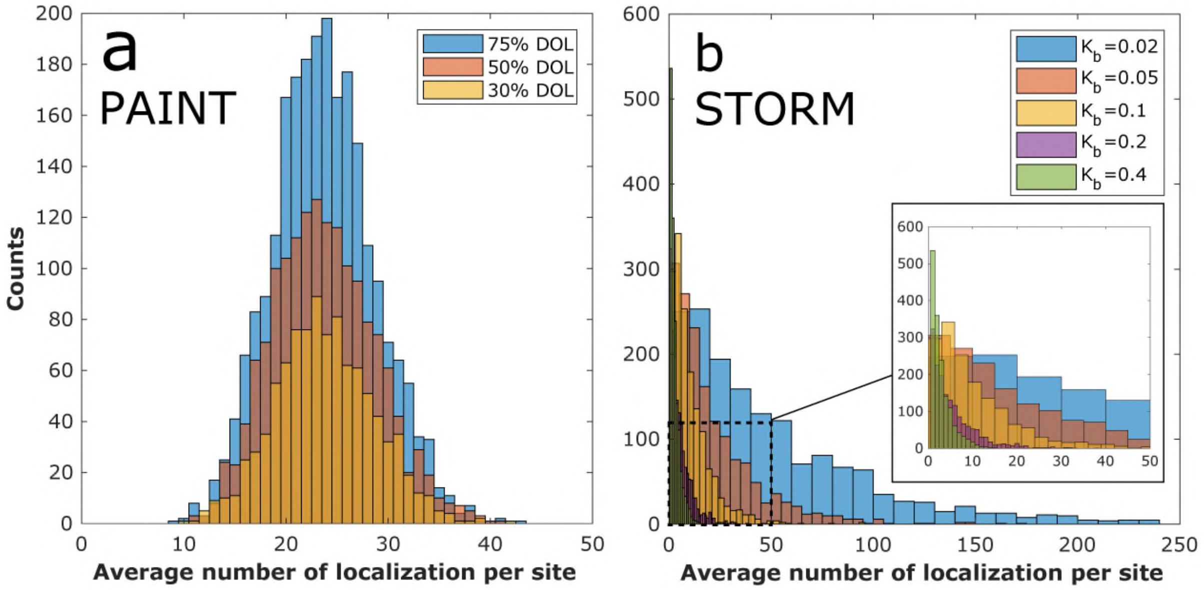

Supplementary Figure 2 | Statistics of localizations per binding sites for (a) PAINT and (b) STORM simulations. For PAINT particles, the distribution of localizations per site follows a Gaussian distribution while for STORM it is a Poisson. In case of STORM data, higher bleaching rate result in fewer localization per sites and a narrower bandwidth for the distribution.

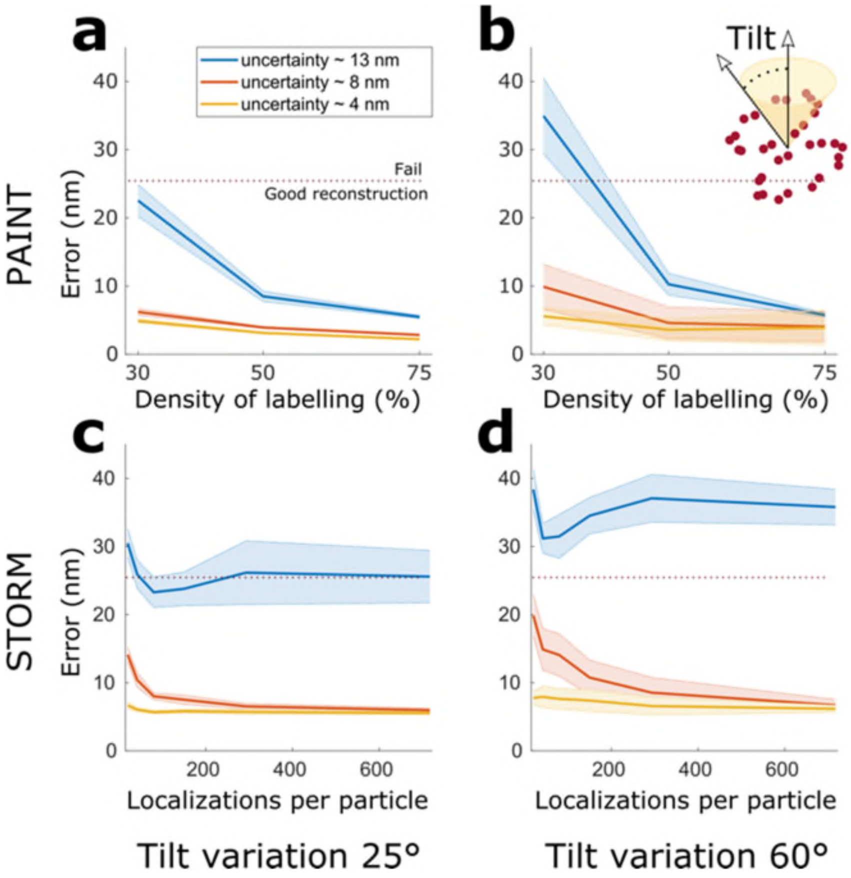

Supplementary Figure 3 | Particle fusion error for the alignment of PAINT and STORM SMLM images of simulated Nup107 particles with different initial tilt variations. (a-b) Particle fusion error of simulated PAINT data for different DOLs and for two range of tilt variations. (c-d) Particle fusion error of simulated STORM data for different number of localizations per particle (proportional to bleaching rate) and for two range of tilt variations. The particle fusion performance is getting slightly worse by increasing the tilt variations but in general it is quite stable even at high tilt angle range of 60 degree. Solid lines indicate the mean and shaded area show the standard error of the mean (n=15).

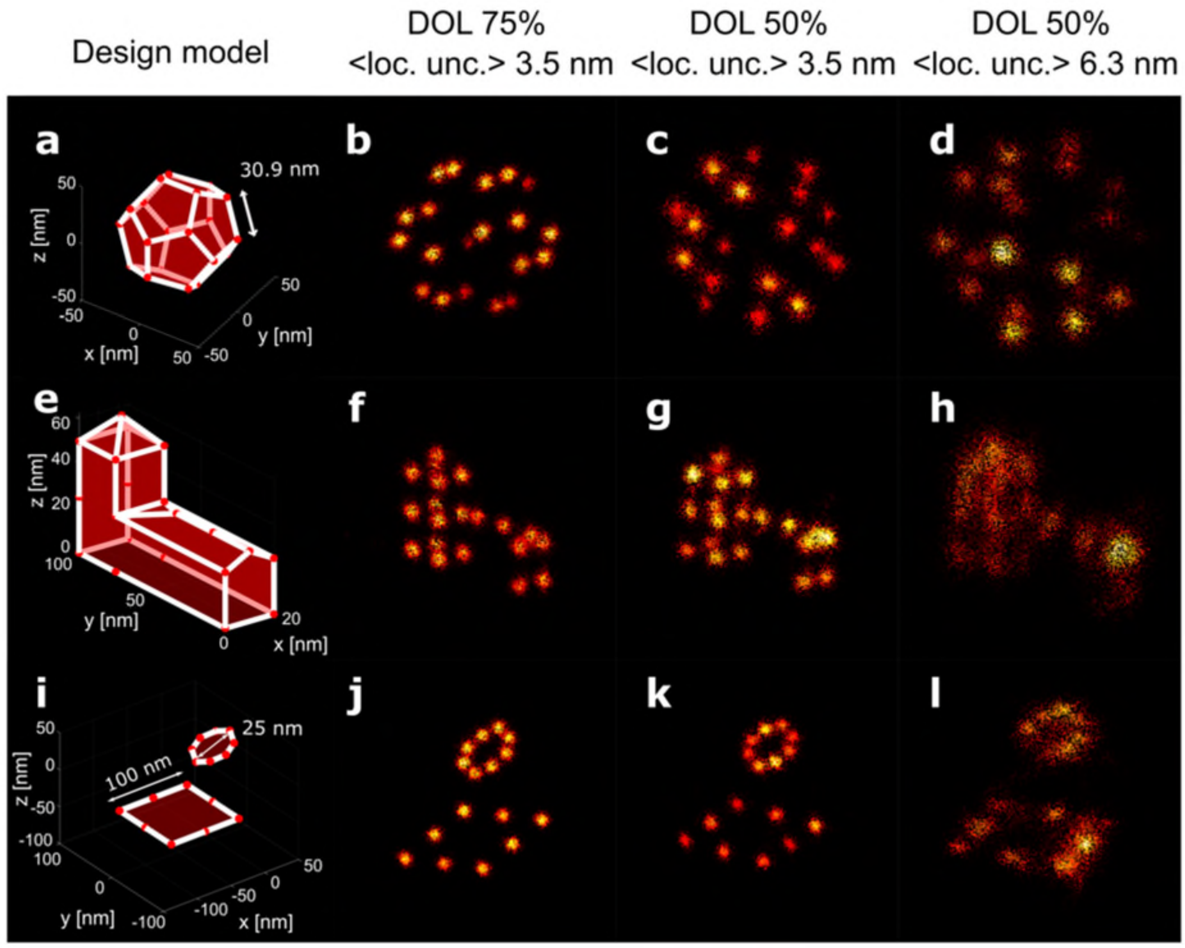

Supplementary Figure 4 | Fusion of 50 simulated particles of three structures with different degree of symmetricalness. (a-d) “Dodecahedron”, (e-h) “building” and (i-l) “ring-square” as examples of a highly symmetric, semi-symmetric and asymmetric structures, respectively. For all cases, 50 particles with arbitrary poses are simulated using PAINT kinetics with DOL of 75% (b, f and j) and 50% (c, g, k) and a photon count of 5000 resulting in an average lateral localization uncertainty of ~3.5 nm. With these settings, the reconstructed superparticles perfectly match the design models in (a, e and i) used for the simulation with fluorophores on the corners (red balls). In case of 50% DOL and mean localization uncertainty ~6.3 nm (d, h, l), the result still resembles the structure, however, with unsharp binding sites at locations where the distance between the binding sites is close to the mean localization uncertainty (the top of the building (h) and the ring above the square (l)).

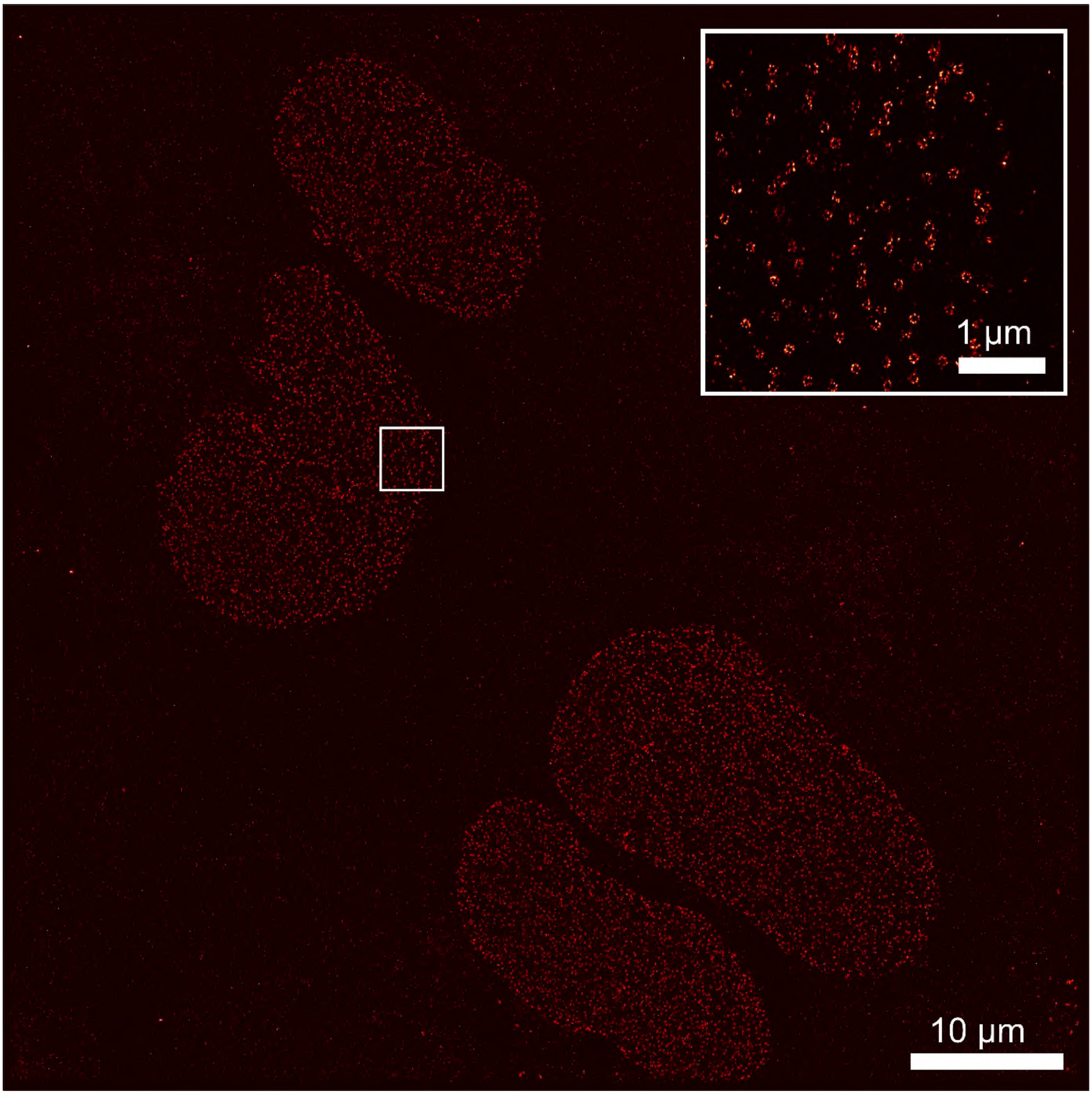

Supplementary Figure 5 | Whole field of view of SNAP-Tag labelled Nup107 proteins for DNA-PAINT imaging. The field of view shows the nuclei of four U2OS cells. The insert in the top-right corner presents a zoom in into the highlighted area. This is a typical result, representative from 5 repeats.

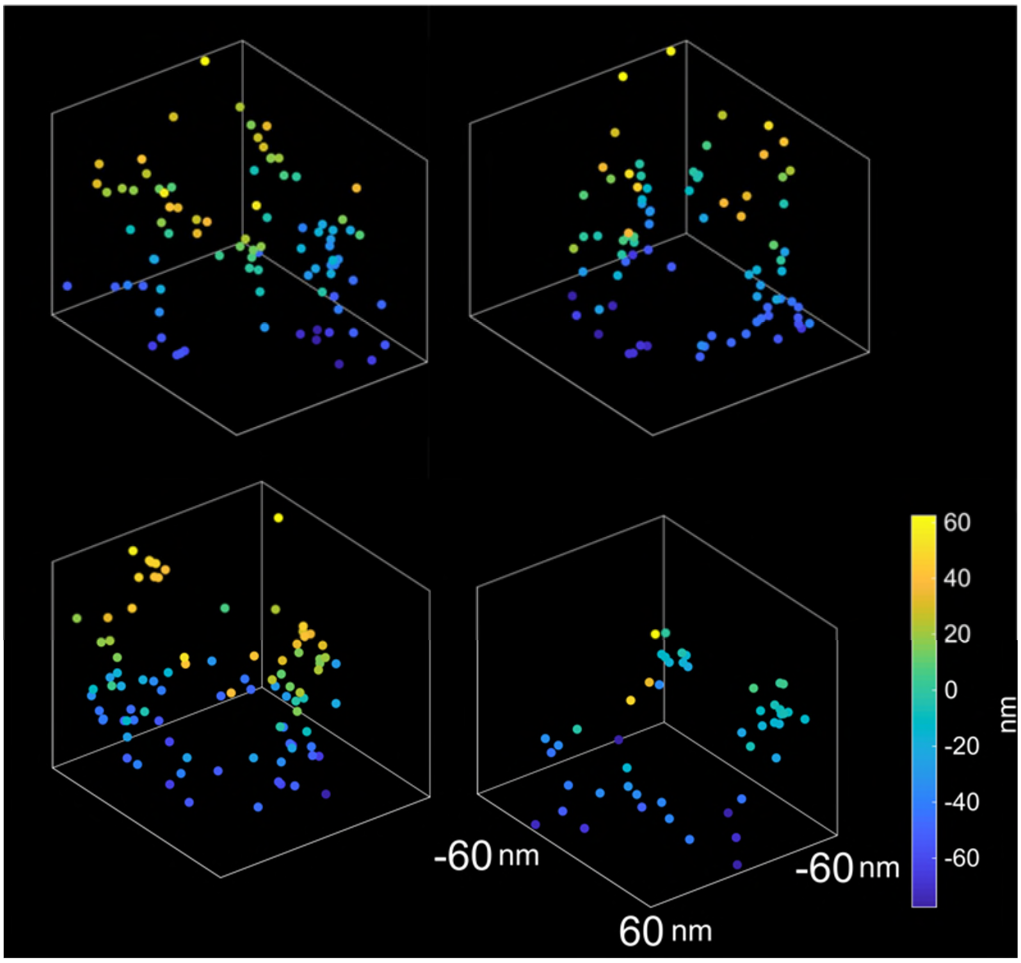

Supplementary Figure 6 | Example images of SNAP-Tag labelled Nup107 particles imaged with PAINT. The individual localizations are color-coded in z. The average number of localizations per particle for this dataset is ~88.

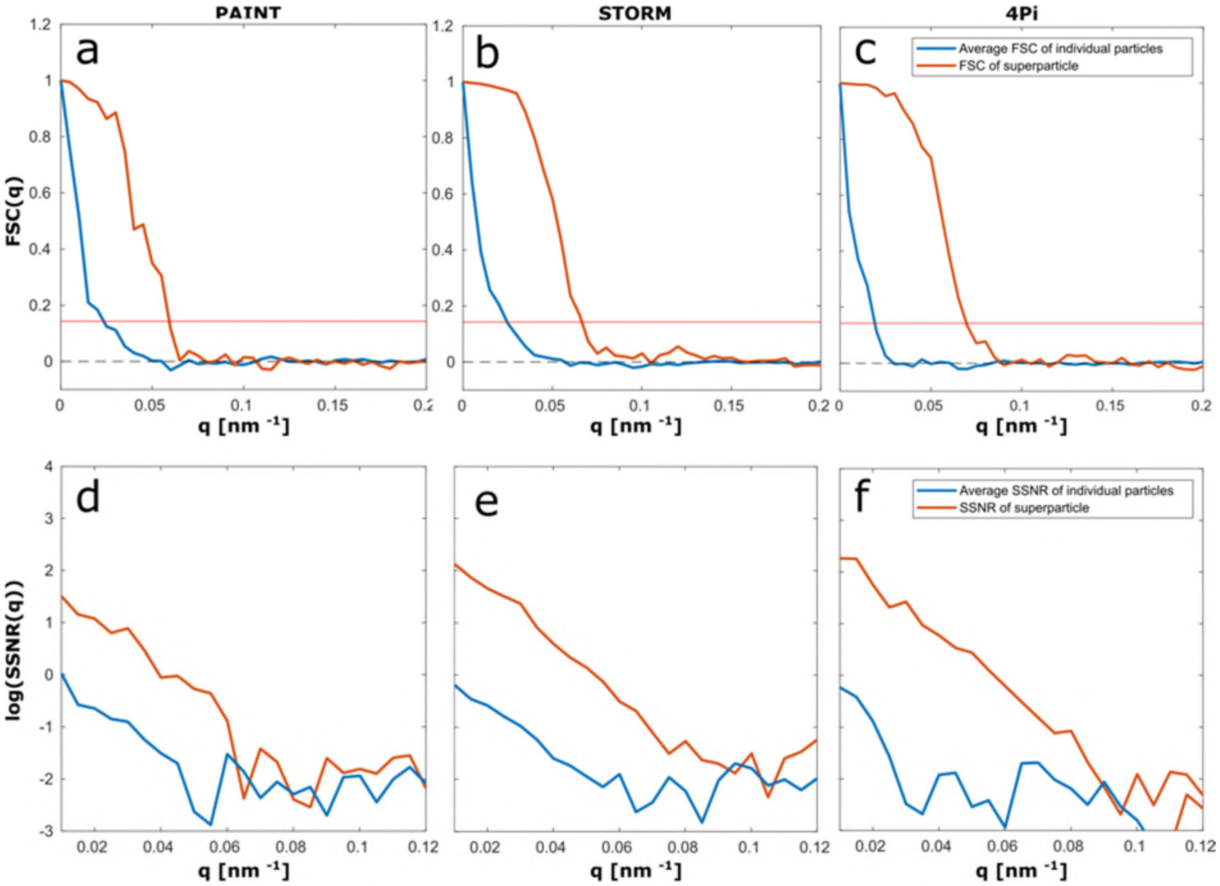

Supplementary Figure 7 | Fourier shell correlation[1] (FSC) and spectral signal-to-noise ratio (SSNR) curves for the initial particles and the corresponding super-particles of 3D astigmatic PAINT, 3D astigmatic STORM and 4Pi STORM data. (a-c) The FSC curves show the resolution improvement from 42.6, 40.5 and 52.2 nm to 16.6, 15.1 and 14.2 nm for the three reconstructions respectively. (d-f) The SSNR curves show about two orders of magnitude improvement in spectral signal-to-noise ratio overall. These values are in good accordance with the visual quality of the super-particles. From these FSC values it is also clear that the dimers cannot be resolved which are at 12 nm distance according to the EM model. The FSC/SSNR curves for particle pairs averages (blue) are computed between pairs of individual particles and then averaged. SSNR is computed as SSNR = FSC/(1-FSC).

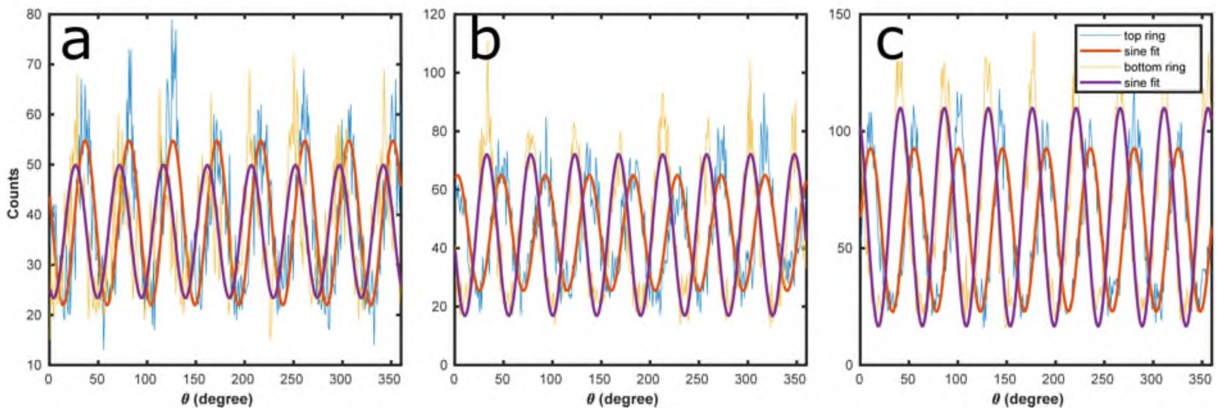

Supplementary Figure 8 | The distribution of the localizations over azimuthal angle and the fitted sine function for the super-particles in Figure 2. (a) PAINT reconstruction. (b) STORM reconstruction. (c) 4Pi reconstruction. In order to find the phase shift between the cytoplasmic and nuclear rings, we fit a sine function to the azimuthal angles of the localization data points in each ring. The difference in the phases of the fitted sine function for each reconstruction defines the azimuthal phase shift of the two rings.

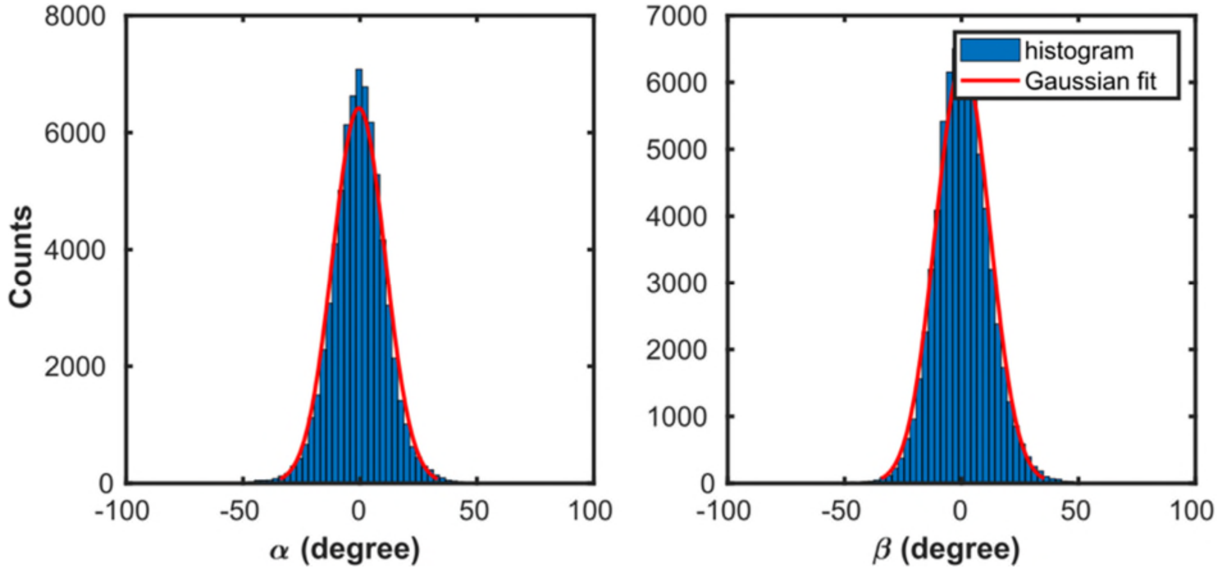

Supplementary Figure 9 | Tilt variations of the STORM experimental Nup107 particles. (a-b) The histograms of the Euler angles α and β (rotation around x and y axis) expressing the tilt variations of the unaligned particles with respect to each other. Both histograms fit a normal distribution with a standard deviation of ~12°.

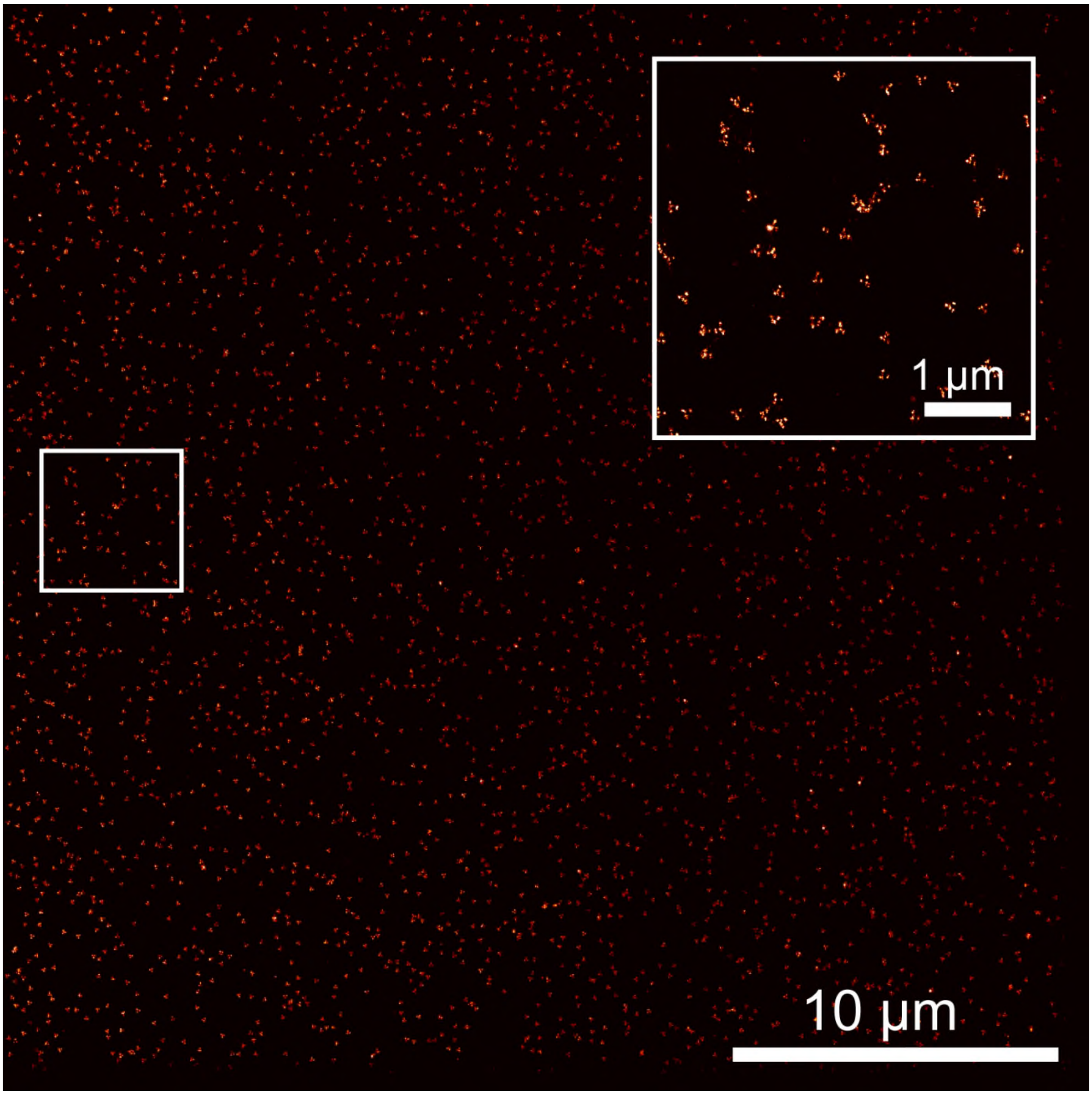

Supplementary Figure 10 | Whole field of view of three-dimensional DNA origami tetrahedron structures imaged with DNA-PAINT on a spinning disk microscope. The side length of the symmetric tetrahedron structure is 100 nm. The insert in the top-right corner presents a zoom in into the highlighted area. This field of view is only a part of a whole cover slip, but more than two independent repeats show similar results.

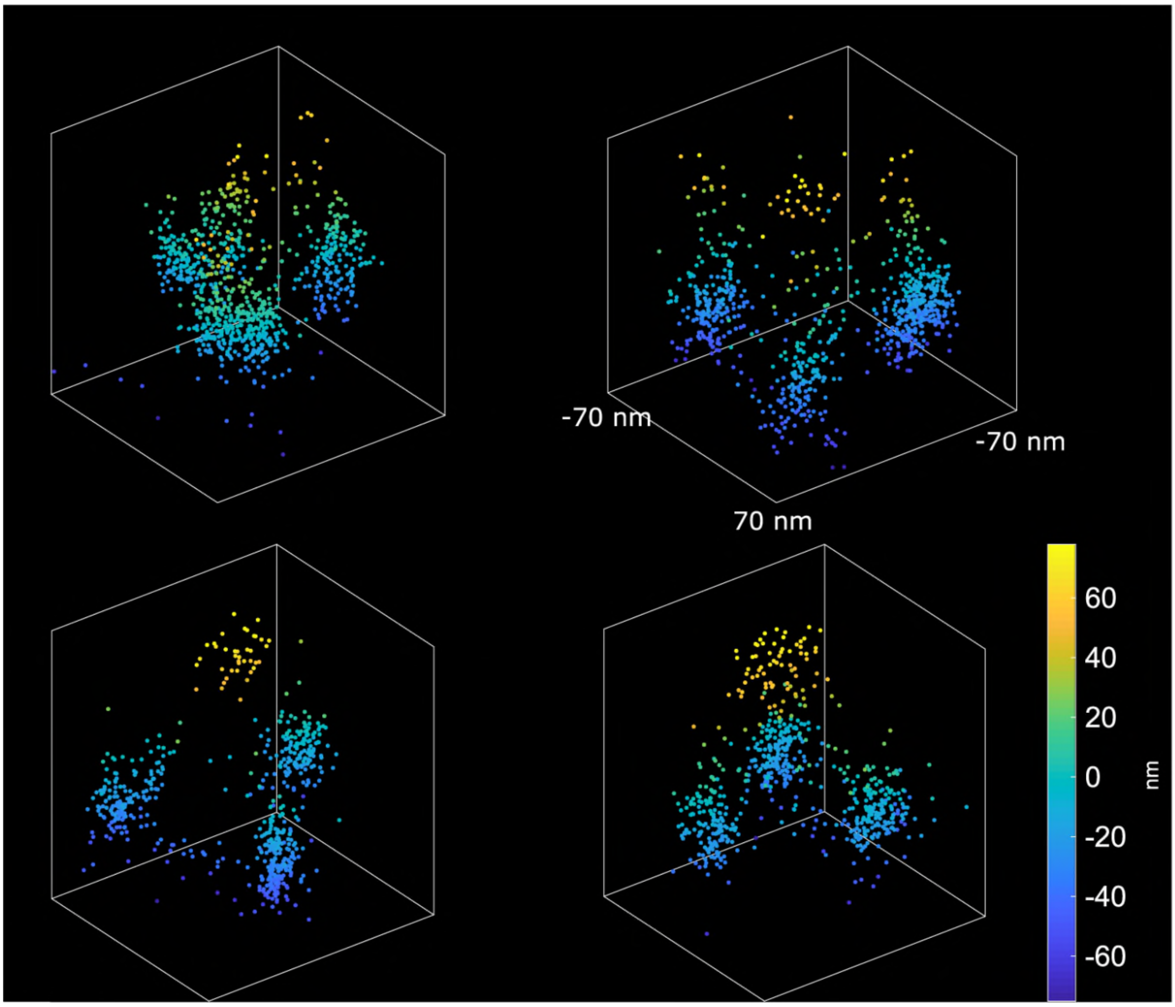

Supplementary Figure 11 | Example images of tetrahedron DNA-origami nanostructures imaged with PAINT.

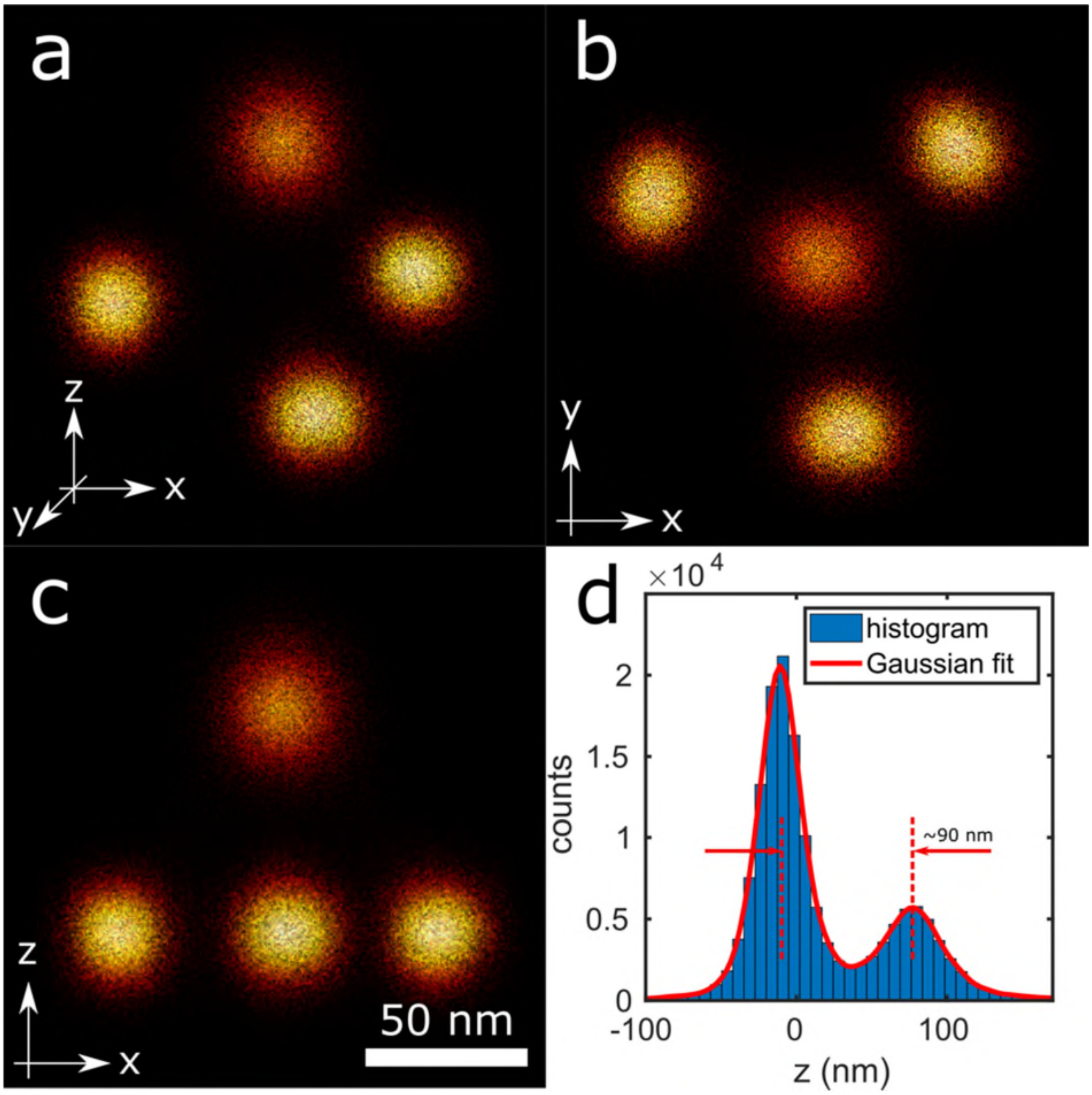

Supplementary Figure 12 | Fusion of 400 tetrahedron DNA-origami nanostructures. (a) Side view of the super-particle. (b) Top (x-y) view of the super-particle. (c) Front (x-z) view of the super-particle. (d) Histogram of the z coordinate of the localization data showing a distance of ~90 nm between the two peaks. This implies a side length of 104 nm which agrees well with the origami design of 100 nm^2. Particle fusion of the nanostructures result in an isotropic distribution of the localization over the four binding sites of the tetrahedron as seen from the round localization distributions around the binding sites.

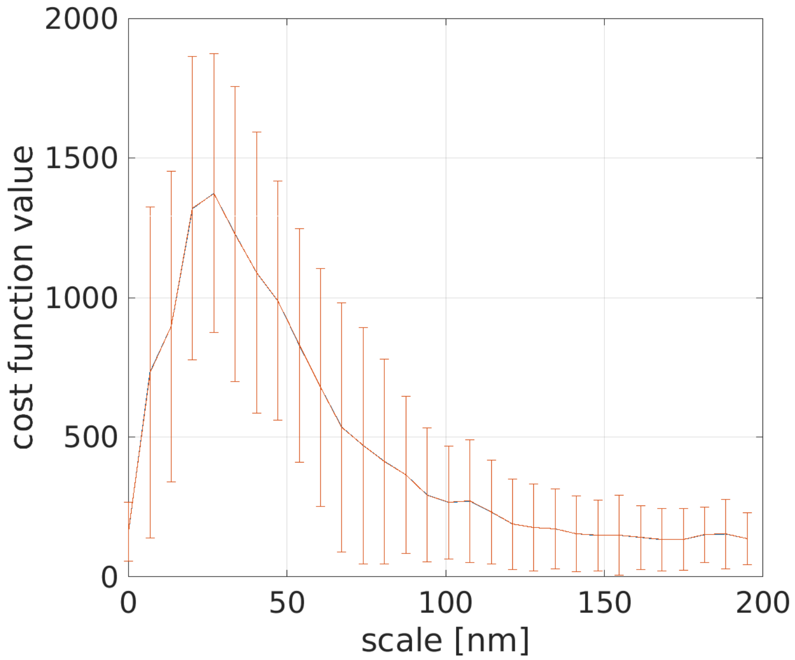

Supplementary Figure 13 | Automatic scale selection for GMM registration. The mean (line) and one standard deviation (error bar) in cost function values for n=10 independent scale sweeps over 50 different particle pairs. The peak of the line indicates the optimal scale parameter which can be subsequently used for the fusion of all particles.

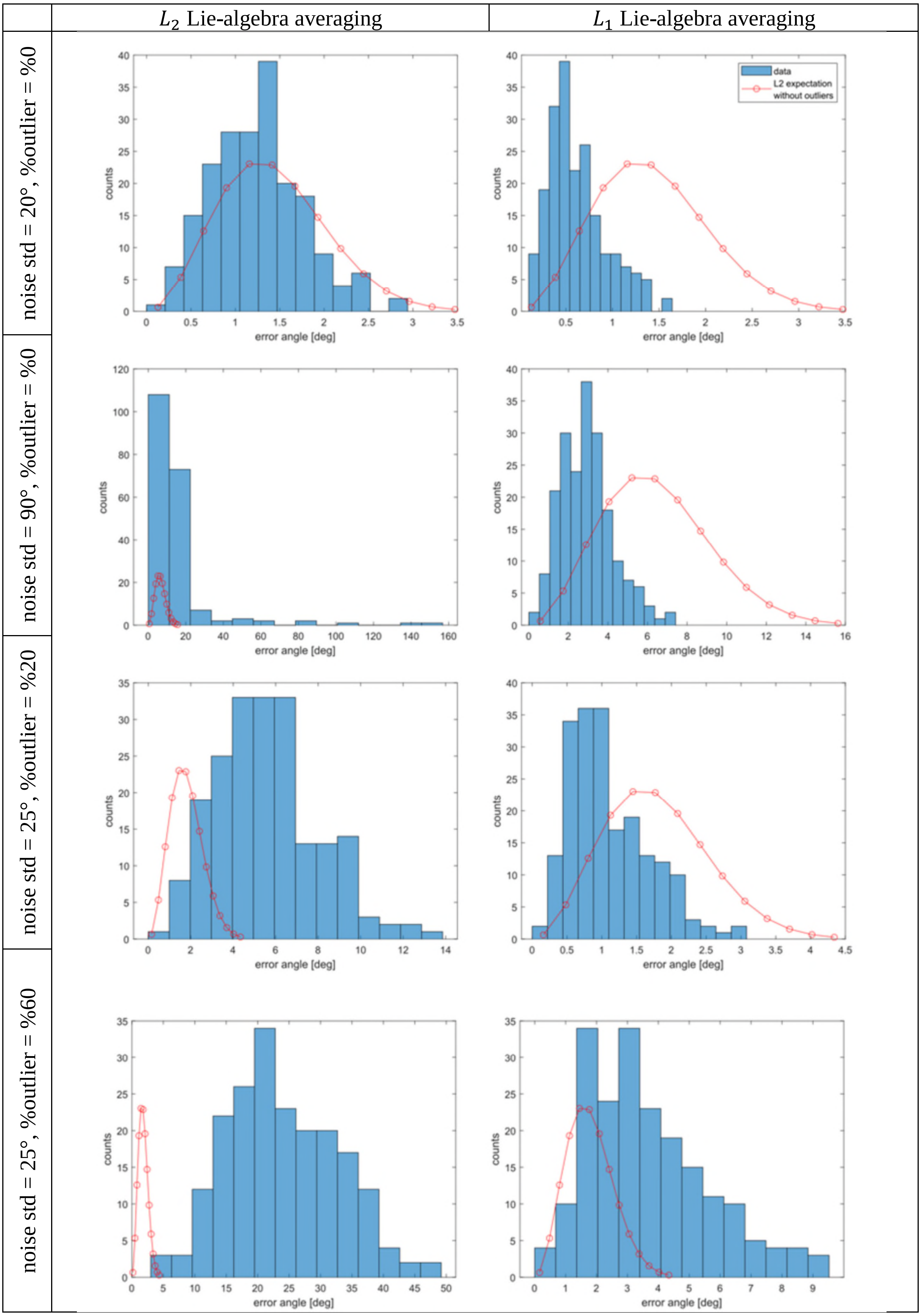

Supplementary Figure 14 | The comparison of the performance of L_2 (left column) and L_1 (right column) Lie-algebraic averaging in the presence of registration error and outliers. Zero mean Gaussian distributed angles are added to ground-truth relative rotations to simulate registration error. For

outliers, a certain percentage of ground-truth relative rotations are replaced by totally random rotations. Each row compares the histogram of the error between the ground-truth rotations and the result of Lie-algebraic averaging with L_1 and L_2 norms together with the expected error (red curve) based on the distribution of the simulated added noise. The graphs suggest that L_2 norm Lie-algebra averaging is very sensitive to the presence of outliers and L_1 norm Lie-algebra averaging outperforms it in all scenarios. Furthermore, L_1 norm Lie-algebra averaging performs better than expected (the histogram is on the left side of the hypothesis curve) due to better handling of outliers for the first three settings.

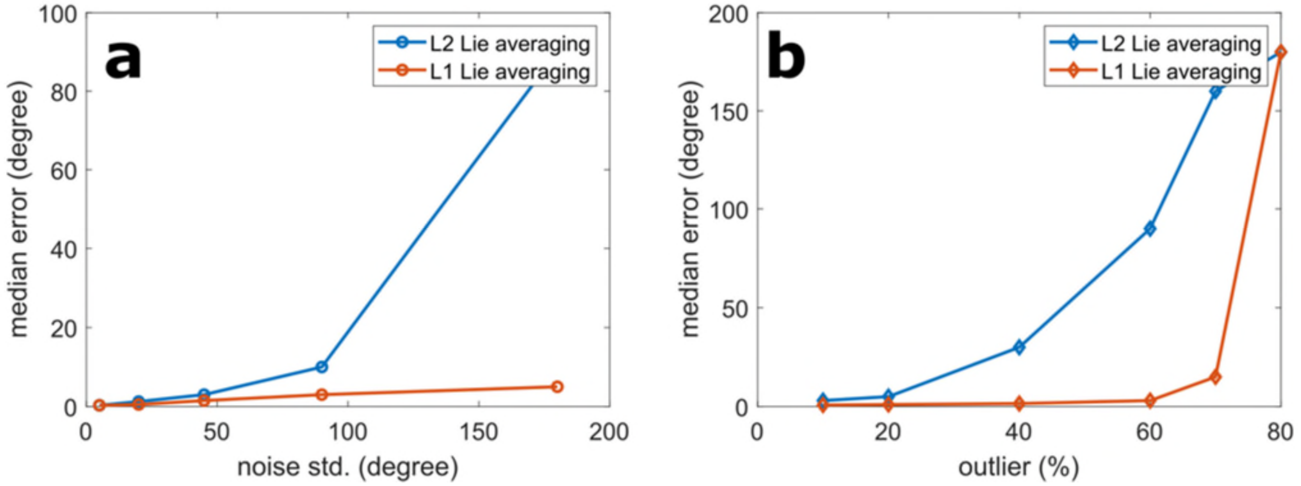

Supplementary Figure 15 | Comparison of the performance of L1 and L2 Lie-algebra averaging (a) as a function of only registration error and (b) as a function of registration outlier percentage, in the presence of a fixed mean registration error of 25 degree.

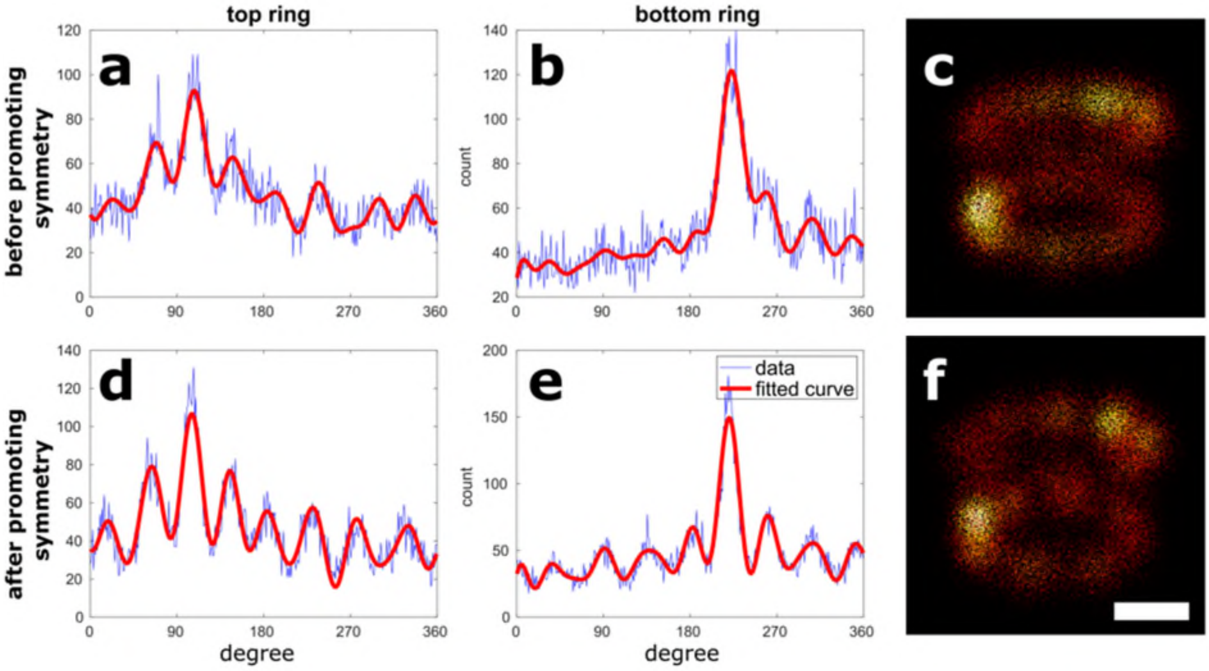

Supplementary Figure 16 | The effect of symmetry promoting on data-driven template for the fusion of 306 PAINT Nup107 particles. (a-b) The distribution of the localizations over azimuthal angle (blue) and the fitted (red) curves for the top and bottom rings of the superparticle in (c) after Lie-algebra averaging and before bootstrapping. (d-e) Similar figure as in (a-b) for the superparticle in (f) after promoting symmetry. Although the 8-fold symmetry of the NUP structure is already revealed in the top ring (a) of the initial superparticle (c), symmetry promotion enhanced the 8-fold symmetry in both rings and subsequently provided a better data-driven template (see Supplementary Movie 5 for 3D visualization). Scale bar is 50 nm.

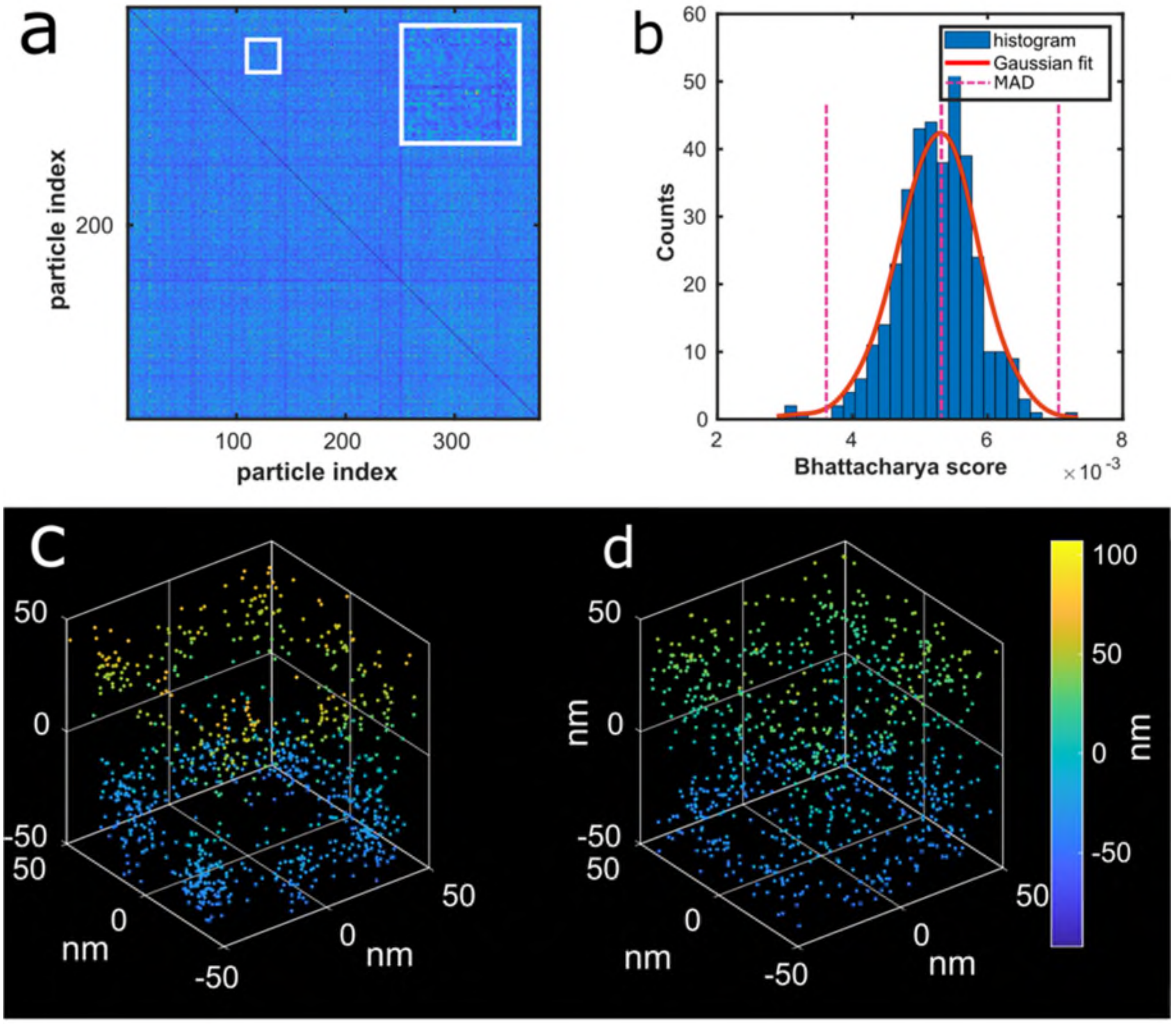

Supplementary Figure 17 | Outlier particle removal. **(a)** All-to-all Bhattacharya heat map matrix for the fusion of 356 particles from the STORM dataset. Each value is rendered as a pixel in a 356×356 image. **(b)** The histogram of the Bhattacharya scores (cost function value) of each particle which is obtained by averaging the matrix in **a** along the columns (or rows) together with the median absolute deviations (MAD) magenta line magenta and its lower and upper bounds. Only 5 particles are recognized as outliers in this dataset with these default settings of the MAD threshold. **(c)** Overlay of the localizations of the best particles (10 top scores). **(d)** Overlay of the localizations of the worst particles (10 lowest scores). While the (good) particles in **c** form a sharp super-particle, the overlay in **d** is quite blurry and the localizations are more scattered around the NUP structure.

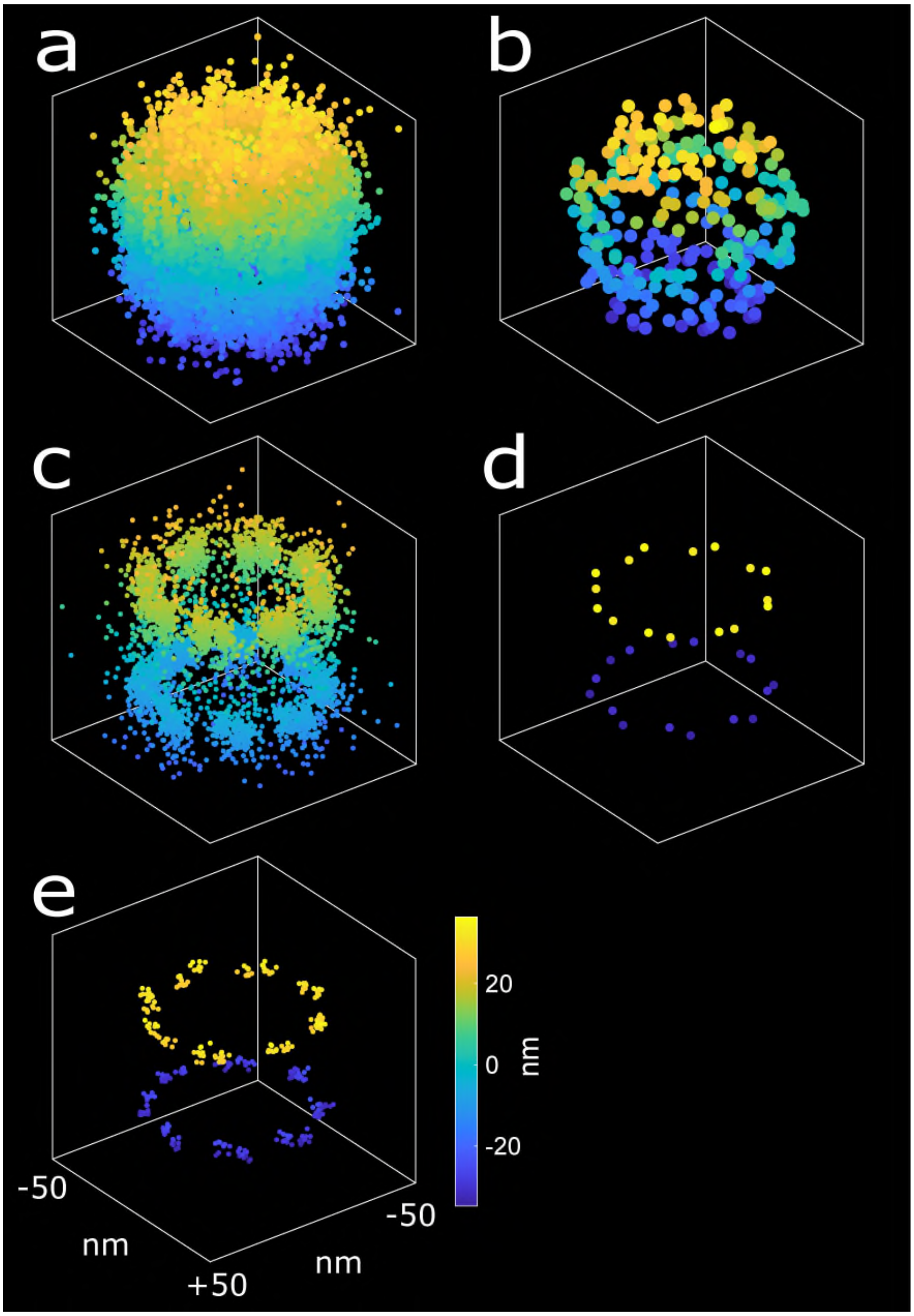

Supplementary Figure 18 | The principle of the proposed registration error measurement. (a) Overlay of 10 simulated particles before alignment. (b) Overlay of the binding sites of the particles in a. (c) Superparticle as a result of fusing the particles in a. (d) The corresponding binding sites of the aligned particles in c in a perfect fusion (zero measurement error). (e) The corresponding binding sites of the aligned particles in c with the effect of the registration error taken into account. Ideally and in a perfect fusion, all the binding sites of the ground-truth simulation model should co-locate. Due to the registration error they scatter around the mean shape model of the super-particle. The corresponding registration error for a run of the particle fusion pipeline is found by quantifying this scatter.

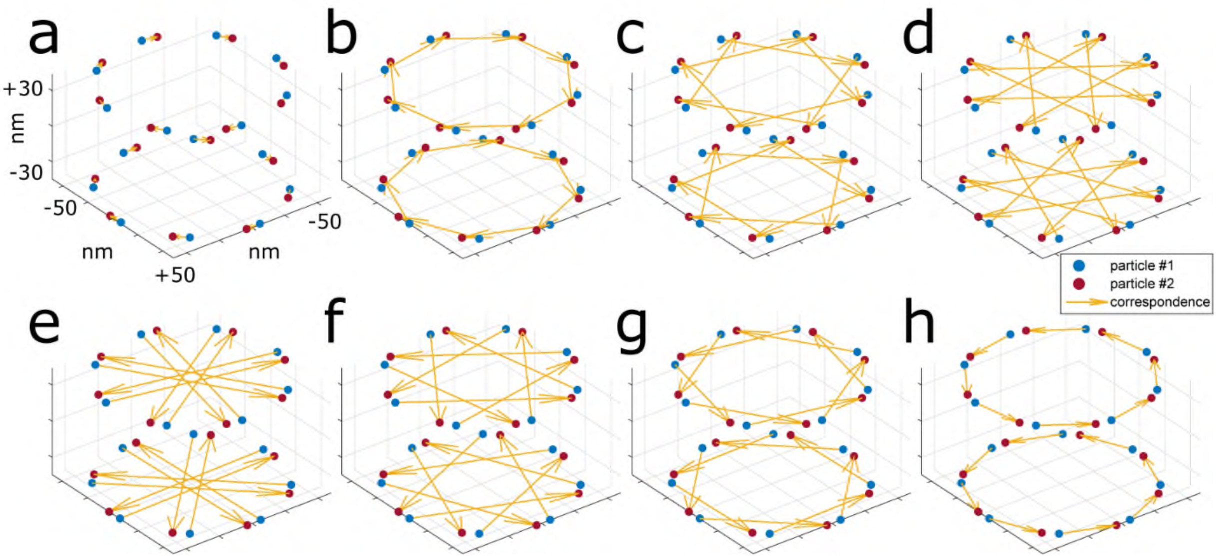

Supplementary Figure 19 | Correspondence problem in matching binding sites of two aligned particles. (a-h) Different correspondence possibilities for computing the error between the registered particles with registration error. In this example, each particle includes 16 binding sites. Since the binding sites are ordered, there are only 8 different combinations of the correspondences between them. The minimum Euclidean distance among these eight candidates defines the correct correspondence and its value is the alignment error.

Supplementary Note 1: Automatic scale selection for GMM registration

We have implemented a pre-alignment step that aims to automatically search for an appropriate scale. The algorithm picks a user selected number of particle pairs (typical value of 50 would suffice) at random from the full dataset and aligns them over a range of scales. The scale for which the average cost function has a peak is then used. This pre-alignment scale sweep step makes it easier and more objective to register novel structures for which the proper scale cannot be known a priori. **Supplementary Figure 13** shows error bars representing the standard deviation for 10 scale sweeps using different particle pairs each time. The cost values are highly variant as a result of the stochastic distribution of the localizations and their uncertainties. It may happen that a particular particle pair is registered worse at the selected optimal scale. All-to-all registration can possibly be improved by allowing a range of scales in addition to the multiple initial rotations. The Bhattacharya cost function would then select the best alignment for each particle pair from every combination of initial rotations and scales. This does however come at the cost of an increase in computation time.

Supplementary Note 2: Proper angular initialization of pair registration

In two-dimensional particle fusion, the pair registration of two particles is initialized with uniform sampling of the $[0, 2\pi]$ interval ($SO(2)$). However, uniform sampling of the three Eulerian angles for the 3D problem does not produce uniform sampling of the Lie-group $SO(3)$. Based on the method that is proposed by Yershova et al. [3], we modified the initialization step of the pair registration in order to properly cover the whole $SO(3)$ landscape for the initial pose of each particle. This method is based on Hopf fibration which describes the 3D rotation group in terms of the circle S^1 and the ordinary 2-sphere S^2. It can be shown that $SO(3)$ is locally isomorphic to the Cartesian product of these two spaces, i.e. $SO(3) \cong S^1 \widetilde{\otimes} S^2$. Multiresolution grids over the circle (m_1) and the 2-sphere (m_2) have $m_1 m_2 2^{3l}$ points at the resolution level l with a base resolution ($l = 0, m_1 = 6, m_2 = 12$) of $m_1 m_2 = 72$ points. The uniform sampling of $SO(3)$ on a grid needs to be done once at each different resolution level. Therefore, these values are stored as a look-up table in the source code of our pipeline and depending on the application the user can invoke a different initialization strategy. It should be noted that increasing the resolution level can lead to excess computational time and therefore it is always recommended to start at the base resolution level.

Supplementary Note 3: L_1 versus L_2 norm Lie-algebraic averaging

All-to-all registration of N given particles provides us with $N(N-1)$ relative transformations R_{ij}^{est} which connect the coordinates in the different poses of particles i and j. Ideally, $R_{ij}^{est} = R_{ij}^{true}$ with

$$R_{ij}^{true} = R_j^{true}\left(R_i^{true}\right)^{-1}, \qquad (1)$$

and R_i^{true} denotes the ground-truth absolute pose of particle i in the global reference frame.

The goal is to find the N individual transformations from a set of given relative transformations. Suppose we have estimates R_i^{est} that are close to the true transformations. Then:

$$S_{ij} = \left(R_j^{est}\right)^{-1} R_{ij}^{true} R_i^{est} \qquad (2)$$

is close to unity, and if we write $R_i^{est} = R_i^{true}(S_i)^{-1}$ then all the S_i are also close to unity. Moreover, we have:

$$S_{ij} = S_j(S_i)^{-1}. \qquad (3)$$

Making use of the logarithmic mapping, the Lie-group elements can be mapped to their equivalent Lie-algebra as follow

$$K_{ij} = \log(S_{ij}) \qquad (4)$$

$$K_i = \log(S_i). \qquad (5)$$

Considering the fact that all S_i and all S_{ij} are close to unity, the Lie-algebra elements K_{ij} and K_i are close to zero, and must satisfy the linear relations:

$$K_{ij} = K_j - K_i \qquad (6)$$

The idea here is that quadratic and higher order terms in the K_i can be neglected, as they are assumed to be small. The pseudo-inverse (least squares) solution of this problem is:

$$K_j = \frac{1}{N}\sum_{i=1}^{N} K_{ij} \qquad (7)$$

up to an arbitrary constant term, representing a global transformation that can always be applied to the collective set of particles. The improved estimate for the individual transformations is then:

$$R_i^{est\prime} = R_i^{est} S_i. \qquad (8)$$

This, in turn, leads to an update of the residual relative transformations:

$$S'_{ij} = \left(R_i^{est\prime}\right)^{-1} R_{ij}^{true} R_j^{est\prime} = (S_i)^{-1} S_{ij} S_j \qquad (9)$$

that should be even closer to unity than S_{ij}, and to $R_i^{est\prime} = R_i^{true}(S'_i)^{-1}$ where the S'_i are even closer to unity than the S_i. These next updates S'_i can be computed along the same lines, etc. The idea of Lie-algebra averaging is to iteratively compute the estimates R_i^{est} by repeatedly solving the linear set of equations for the Lie-algebra elements of the update transformations for the individual transformation estimates. The final overall estimate of the individual transformations is:

$$R_i^{est,final} = R_i^{est,initial} S_i S'_i S''_i \ \ldots \qquad (10)$$

It is assumed that this procedure will converge to the correct result, i.e. that $R_i^{est,final} = R_i^{true}$, but this is by no means obvious.

Alternative to the least square solution above (which corresponds to the L_2 norm), we can reformulate the linear system of equations in equation 6 to solve it with the L_1 norm, which appears to be more robust to outlier registrations. In this approach, we try to solve the following minimization problem:

$$\min_{\boldsymbol{x}} \|A\boldsymbol{x} - \boldsymbol{b}\|_1 \qquad (11)$$

where A is the $N(N-1)/2 \times N$ indicator matrix consisting of -1, 0 and 1 elements, $\boldsymbol{x}$ is the $N \times 1$ column vector of absolute Lie-algebras (K_i) and $\boldsymbol{b}$ is the $N(N-1)/2 \times 1$ vector containing the relative Lie-algebras (K_{ij}).

In order to compare the performance of both approaches, we first simulated 200 NPC particles with completely random orientations (R_i^{gt}). Then, we added a certain amount of noise to their relative poses using

$$R_{ij} = R_i^{gt} R_{noise} (R_j^{gt})^T \qquad (12)$$

where R_{noise} is a rotation matrix with a completely random rotation axis and a rotation angle that is drawn from a zero mean Gaussian distribution with a standard deviation of σ_α. Furthermore, we simulated the effect of completely wrong registrations (outliers), by replacing a fraction of ground-truth relative transformations ($R_{ij}^{gt} = R_i^{gt}(R_j^{gt})^T$) with completely random rotation matrices. Once the simulated noise and outliers are added to the relative ground-truth transformations, we use the L_1 and L_2 Lie-algebra averaging to find the absolute pose of each particle (R_i^{est}). Using equation (2), we can finally compute the resulting estimation error by computing the rotation angle of the matrix S_{ij} as follows

$$|\theta_{ij}| = \arccos\left(\frac{\text{Trace}(S_{ij}) - 1}{2}\right). \qquad (13)$$

In **Supplementary Figure 14**, we show the histograms of θ_{ij} at different noise and outlier settings to show how L_1 Lie-algebra averaging outperforms L_2 Lie-algebra averaging especially for large amounts of outlier registrations. **Supplementary Figure 15** summarizes the comparison for a range of values for noise and outlier percentages.

References

1. Nieuwenhuizen, R.P. et al. Measuring image resolution in optical nanoscopy. *Nat Methods* **10**, 557-562 (2013).
2. Schueder, F. et al. Multiplexed 3D super-resolution imaging of whole cells using spinning disk confocal microscopy and DNA-PAINT. *Nat Comm* **8**, 2090 (2017).
3. Yershova A, Jain S, Lavalle SM, Mitchell JC. Generating uniform incremental grids on SO(3) using the Hopf fibration. *Int J Rob Res*. 2010;29(7):801-812.
