# Supplementary material for: 3D particle averaging and detection of macromolecular symmetry in localization microscopy
Source: Nat Commun. 2021 May 14;12:2847. doi: 10.1038/s41467-021-22006-5 (PMC8121824; doi:10.1038/s41467-021-22006-5)
Supplement: Supplementary file 2 — Description of Additional Supplementary Files [file 41467_2021_22006_MOESM2_ESM.pdf]

Title: Supplementary Movie 1
Description: 3D visualization of the superparticle as a result of fusing 306 experimental Nup107 PAINT particles.

Title: Supplementary Movie 2
Description: 3D visualization of the superparticle as a result of fusing 356 experimental Nup107 STORM particles.

Title: Supplementary Movie 3
Description: 3D visualization of the superparticle as a result of fusing 300 experimental Nup96 STORM particles together with its detected symmetry axis.

Title: Supplementary Movie 4
Description: 3D visualization of the superparticle as a result of fusing 400 experimental Tetrahedron DNA-origami nanostructures imaged using PAINT together with its detected symmetry axes. The white bars show the 3-fold symmetry axes of the tetrahedron.

Title: Supplementary Movie 5
Description: The effect of symmetry promotion on the superparticle of Nup107 PAINT data after Lie-algebra averaging step.
