# Supplementary material for: 3D particle averaging and detection of macromolecular symmetry in localization microscopy
Source: Nat Commun. 2021 May 14;12:2847. doi: 10.1038/s41467-021-22006-5 (PMC8121824; doi:10.1038/s41467-021-22006-5)
Supplement: Supplementary file 4 — Reporting Summary [file 41467_2021_22006_MOESM4_ESM.pdf]

nature research

Corresponding author(s): Bernd Rieger

Last updated by author(s): Feb 11, 2021

Reporting Summary

Nature Research wishes to improve the reproducibility of the work that we publish. This form provides structure for consistency and transparency in reporting. For further information on Nature Research policies, see our Editorial Policies and the Editorial Policy Checklist.

Statistics

For all statistical analyses, confirm that the following items are present in the figure legend, table legend, main text, or Methods section.

n/a	Confirmed	
☐	☒	The exact sample size (n) for each experimental group/condition, given as a discrete number and unit of measurement
☐	☒	A statement on whether measurements were taken from distinct samples or whether the same sample was measured repeatedly
☒	☐	The statistical test(s) used AND whether they are one- or two-sided<br>*Only common tests should be described solely by name; describe more complex techniques in the Methods section.*
☒	☐	A description of all covariates tested
☒	☐	A description of any assumptions or corrections, such as tests of normality and adjustment for multiple comparisons
☐	☒	A full description of the statistical parameters including central tendency (e.g. means) or other basic estimates (e.g. regression coefficient) AND variation (e.g. standard deviation) or associated estimates of uncertainty (e.g. confidence intervals)
☒	☐	For null hypothesis testing, the test statistic (e.g. F, t, r) with confidence intervals, effect sizes, degrees of freedom and P value noted<br>*Give P values as exact values whenever suitable.*
☒	☐	For Bayesian analysis, information on the choice of priors and Markov chain Monte Carlo settings
☒	☐	For hierarchical and complex designs, identification of the appropriate level for tests and full reporting of outcomes
☒	☐	Estimates of effect sizes (e.g. Cohen's d, Pearson's r), indicating how they were calculated

Our web collection on statistics for biologists contains articles on many of the points above.

Software and code

Policy information about availability of computer code

Data collection	The code implementation (on CPU and GPU) is a major part of the research as it concerns an algorithm. The source code is available under our public Github repository at https://github.com/imphys/smlm_datafusion3d. The data was collected on custom-made microscopes. The used software include fit3Dcspline (https://github.com/jries/fit3Dcspline) and Picasso (https://github.com/jungmannlab/picasso) for STORM and PAINT data, respectively.
Data analysis	STORM modality data were analyzed using fit3Dcspline (https://github.com/jries/fit3Dcspline). PAINT data were processed using software package Picasso (https://github.com/jungmannlab/picasso). The 3D particle fusion code of the publication is publicly available via github (https://github.com/imphys/smlm_datafusion3d with DOI: https://doi.org/10.4121/13743703)

For manuscripts utilizing custom algorithms or software that are central to the research but not yet described in published literature, software must be made available to editors and reviewers. We strongly encourage code deposition in a community repository (e.g. GitHub). See the Nature Research guidelines for submitting code & software for further information.

Data

Policy information about availability of data

All manuscripts must include a data availability statement. This statement should provide the following information, where applicable:

- Accession codes, unique identifiers, or web links for publicly available datasets
- A list of figures that have associated raw data
- A description of any restrictions on data availability

The NUP107 and tetrahedron 3D localization data are available at: https://doi.org/10.4121/13797686
The NUP96 3D localization data are available at: https://www.ebi.ac.uk/biostudies/BioImages/studies/S-BIAD8

Field-specific reporting

Please select the one below that is the best fit for your research. If you are not sure, read the appropriate sections before making your selection.

☒ Life sciences ☐ Behavioural & social sciences ☐ Ecological, evolutionary & environmental sciences

For a reference copy of the document with all sections, see nature.com/documents/nr-reporting-summary-flat.pdf

Life sciences study design

All studies must disclose on these points even when the disclosure is negative.

Sample size	We performed simulations and tests on DNA origami and the nuclear pore complex. For the NPC, hundreds of NPCs were imaged with little variability. NPCs were pooled from different cells on different coverslips.
Data exclusions	Only data from experiments that obviously failed (wrong microscope setup/out-of-focus/sample staining/wrong emitter activation/failed drift correction/failed 3D registration) were excluded.
Replication	All experiments were reliably produced and all attempts at replication were successful. Nmmber of repeats are mentioned in the captions were applicable.
Randomization	Randomization is not applicable.
Blinding	Blinding was not done.

Reporting for specific materials, systems and methods

We require information from authors about some types of materials, experimental systems and methods used in many studies. Here, indicate whether each material, system or method listed is relevant to your study. If you are not sure if a list item applies to your research, read the appropriate section before selecting a response.

Materials & experimental systems

n/a	Involved in the study	
☒	☐	Antibodies
☐	☒	Eukaryotic cell lines
☒	☐	Palaeontology and archaeology
☒	☐	Animals and other organisms
☒	☐	Human research participants
☒	☐	Clinical data
☒	☐	Dual use research of concern

Methods

n/a	Involved in the study	
☒	☐	ChIP-seq
☒	☐	Flow cytometry
☒	☐	MRI-based neuroimaging

Eukaryotic cell lines

Policy information about cell lines

Cell line source(s)	U2OS Nup107-SNAP-tag (catalog no. 300294). Cell line is available from CLS Cell Line Service, Eppelheim, Germany.
Authentication	Cell lines were not further authenticated.
Mycoplasma contamination	Cell lines have not been tested for mycoplasma contamination
Commonly misidentified lines (See ICLAC register)	No commonly misidentified cell lines were used.
